# Argonaute 2 modulates EGFR–RAS signaling to promote mutant *HRAS* and *NRAS-*driven malignancies

**DOI:** 10.1093/pnasnexus/pgac084

**Published:** 2022-07-28

**Authors:** Ronald F Siebenaler, Seema Chugh, Jessica J Waninger, Vijaya L Dommeti, Carson Kenum, Malay Mody, Anudeeta Gautam, Nidhi Patel, Alec Chu, Pushpinder Bawa, Jennifer Hon, Richard D Smith, Heather Carlson, Xuhong Cao, John J G Tesmer, Sunita Shankar, Arul M Chinnaiyan

**Affiliations:** Michigan Center for Translational Pathology, University of Michigan, Ann Arbor, MI 48109, USA; Department of Pathology, University of Michigan, Ann Arbor, MI 48109, USA; Michigan Center for Translational Pathology, University of Michigan, Ann Arbor, MI 48109, USA; Department of Pathology, University of Michigan, Ann Arbor, MI 48109, USA; Michigan Center for Translational Pathology, University of Michigan, Ann Arbor, MI 48109, USA; Department of Pathology, University of Michigan, Ann Arbor, MI 48109, USA; Michigan Center for Translational Pathology, University of Michigan, Ann Arbor, MI 48109, USA; Department of Pathology, University of Michigan, Ann Arbor, MI 48109, USA; Michigan Center for Translational Pathology, University of Michigan, Ann Arbor, MI 48109, USA; Department of Pathology, University of Michigan, Ann Arbor, MI 48109, USA; Michigan Center for Translational Pathology, University of Michigan, Ann Arbor, MI 48109, USA; Department of Pathology, University of Michigan, Ann Arbor, MI 48109, USA; Michigan Center for Translational Pathology, University of Michigan, Ann Arbor, MI 48109, USA; Department of Pathology, University of Michigan, Ann Arbor, MI 48109, USA; Michigan Center for Translational Pathology, University of Michigan, Ann Arbor, MI 48109, USA; Department of Pathology, University of Michigan, Ann Arbor, MI 48109, USA; Michigan Center for Translational Pathology, University of Michigan, Ann Arbor, MI 48109, USA; Department of Pathology, University of Michigan, Ann Arbor, MI 48109, USA; Michigan Center for Translational Pathology, University of Michigan, Ann Arbor, MI 48109, USA; Department of Pathology, University of Michigan, Ann Arbor, MI 48109, USA; Michigan Center for Translational Pathology, University of Michigan, Ann Arbor, MI 48109, USA; Department of Pathology, University of Michigan, Ann Arbor, MI 48109, USA; College of Pharmacy, University of Michigan, Ann Arbor, MI 48109, USA; College of Pharmacy, University of Michigan, Ann Arbor, MI 48109, USA; Michigan Center for Translational Pathology, University of Michigan, Ann Arbor, MI 48109, USA; Department of Pathology, University of Michigan, Ann Arbor, MI 48109, USA; Howard Hughes Medical Institute, University of Michigan, Ann Arbor, MI 48109, USA; Departments of Biological Sciences and Medicinal Chemistry and Molecular Pharmacology, Purdue University, West Lafayette, IN 47907, USA; Michigan Center for Translational Pathology, University of Michigan, Ann Arbor, MI 48109, USA; Department of Pathology, University of Michigan, Ann Arbor, MI 48109, USA; Michigan Center for Translational Pathology, University of Michigan, Ann Arbor, MI 48109, USA; Department of Pathology, University of Michigan, Ann Arbor, MI 48109, USA; Howard Hughes Medical Institute, University of Michigan, Ann Arbor, MI 48109, USA; Department of Urology, University of Michigan, Ann Arbor, MI 48109, USA; Rogel Cancer Center, University of Michigan, Ann Arbor, MI 48109, USA

**Keywords:** HRAS, NRAS, EGFR, Argonaute 2, senescence

## Abstract

Activating mutations in RAS GTPases drive nearly 30% of all human cancers. Our prior work described an essential role for Argonaute 2 (AGO2), of the RNA-induced silencing complex, in mutant *KRAS*-driven cancers. Here, we identified a novel endogenous interaction between AGO2 and RAS in both wild-type (WT) and mutant *HRAS*/*NRAS* cells. This interaction was regulated through EGFR-mediated phosphorylation of Y393-AGO2, and utilizing molecular dynamic simulation, we identified a conformational change in pY393-AGO2 protein structure leading to disruption of the RAS binding site. Knockdown of *AGO2* led to a profound decrease in proliferation of mutant *HRAS*/*NRAS*-driven cell lines but not WT *RAS* cells. These cells demonstrated oncogene-induced senescence (OIS) as evidenced by β-galactosidase staining and induction of multiple downstream senescence effectors. Mechanistically, we discovered that the senescent phenotype was mediated via induction of reactive oxygen species. Intriguingly, we further identified that loss of AGO2 promoted a novel feed forward pathway leading to inhibition of the PTP1B phosphatase and activation of EGFR–MAPK signaling, consequently resulting in OIS. Taken together, our study demonstrates that the EGFR–AGO2–RAS signaling axis is essential for maintaining mutant *HRAS* and *NRAS*-driven malignancies.

Significance Statement RAS proteins (HRAS, NRAS, and KRAS) act as molecular switches to promote cell proliferation, and constitutively active mutations act to drive oncogenesis and growth across multiple cancer lineages. Despite years of research, direct inhibitors of mutant RAS proteins remain elusive for most mutations. In an effort to uncover potential therapeutic targets, our lab previously described an interaction between Argonaute 2 (AGO2) and KRAS, playing an important role in regulating RAS signaling and promoting mutant *KRAS*-driven cancers. Here, we describe a role for AGO2 in mutant *HRAS* and *NRAS*-driven cancers with loss of *AGO2* leading to impaired growth, senescence, and altered RAS signaling. Our results suggest that the AGO2–RAS interaction may be a potential therapeutic target in mutant *RAS-*driven cancers.

## Introduction

RAS GTPases act as growth factor receptor-regulated molecular switches, modulating cellular growth, survival, and differentiation. RAS proteins cycle through nucleotide loading of active GTP-bound and inactive GDP-bound states, and regulator proteins, such as GTPase activating proteins (GAPs) or guanine exchange factors (GEFs), promote the nucleotide exchange at the plasma membrane under the control of EGFR and other growth factor receptors (1–4). Activating mutations in *KRAS, HRAS*, and *NRAS* occur in over 30% of all cancers, with particular prevalence in pancreatic, melanoma, multiple myeloma, and colon cancers (2, 5, 6). Oncogenic mutations in *RAS* inhibit intrinsic GTPase activity, leading to constitutively active RAS signaling independent of growth factor receptor control driving cell transformation (1, 2, 7). Despite our understanding of the signaling events triggered by oncogenic RAS, targeting RAS clinically remains a particularly challenging prospect (3, 8).

With the goal of targeting mutant *KRAS* signaling and identifying novel partners of mutant *RAS*-mediated oncogenesis, we recently probed a panel of human cancer cell lines for protein partners of RAS and identified an interaction between KRAS and Argonaute 2 (AGO2) (9, 10), a key member of the RNA-induced silencing complex (RISC). Specifically, the Switch II domain of KRAS was shown to bind to the N-terminus of AGO2. While this interaction was observed in both wild-type (WT) and mutant *KRAS*-expressing cell lines, we found that AGO2 was required for oncogenic *KRAS*-driven cellular transformation. AGO2’s RISC activity and miRNA duplex unwinding were inhibited by interaction with mutant KRAS (9), suggesting that the RAS–AGO2 interaction plays a dynamic role in promoting mutant *KRAS*-driven cancer. In addition, we recently extended these initial observations in a mutant *Kras*-driven mouse model of pancreatic ductal adenocarcinoma (PDAC) with co-knockout of *Ago2* (11). Furthermore, *Ago2* ablation in a mutant *Kras-*driven nonsmall cell lung cancer (NSCLC) mouse model significantly reduced tumor burden and altered downstream Kras signaling (12). These combined studies demonstrated an important role for the AGO2–RAS interaction in promoting a *KRAS*-driven oncogenic state.

Despite the evidence for a functional role of mutant KRAS–AGO2 interaction in cellular transformation and proliferation, the role of AGO2 in mutant *HRAS* or *NRAS* cancers is unclear. Mutations in *HRAS* and *NRAS* account for approximately 4% and 11% of all *RAS*-driven cancers, respectively (6). Using several cellular models of mutant *HRAS* and *NRAS-*driven cancers, this study evaluated the interaction of AGO2 with these RAS isoforms and elucidated the functional implications of this interaction. Taken together, our results demonstrate an important EGFR–AGO2–RAS signaling axis that plays a key role in mutant *RAS*-driven proliferation, and both mutant HRAS and NRAS depend on AGO2 to overcome senescence.

## Materials and Methods

### Cell culture, transfection, and epidermal growth factor stimulation

All cell lines (detailed in Table S1, Supplementary Material) were obtained from the American Type Culture Collection (ATCC) or DSMZ-German Collection of Microorganisms and Cell Cultures (DSMZ; Kasumi-2). Cells were cultured following ATCC culture methods in media supplemented with the corresponding serum and antibiotics. Additionally, cells were routinely genotyped and tested biweekly for mycoplasma contamination. For epidermal growth factor (EGF) stimulation, cells were grown to approximately 80% confluence and washed with PBS three times. Cells were incubated overnight (16 hours) in serum-free media. EGF stimulation was performed for 5 minutes with 100 ng/µl of EGF (Gibco) at 37°C. After stimulation, cells were washed, and protein lysates were prepared in K Buffer lysis buffer.

U2OS were transfected with different *AGO2* constructs using Fugene HD (Promega) according to manufacturers’ protocols. For EGFR stimulation with transient *AGO2* construct overexpression, cells were transfected approximately 16 hours prior to overnight serum starvation and EGF stimulation.

### Immunoprecipitation and immunoblot analysis

For Immunoprecipitation (IP) analysis, protein lysates were prepared in K Buffer (20 mM Tris pH 7.0, 5 mM EDTA, 150 mM NaCl, 1% Triton X100, 1 mM DTT, phosphatase inhibitors, and protease inhibitors). Typically,150 to 200 µg of protein lysates (RAS10 IP: 150 µg; AGO2 IP: 200 µg; and KRAS IP: 150 µg) were precleared with 10 µl of protein A/G agarose beads (Santa Cruz) for 1 hour. Precleared lysates were incubated with 5 to 10 µg of the indicated primary antibodies targeting the protein of interest or with corresponding isotype controls overnight at 4°C. A volume of 30 µl of protein A/G beads were then added to immune complexes and incubated for 1 to 3 hours at 4°C, spun, and washed in 150 to 300 mM NaCl-containing K Buffer prior to separation of immunoprecipitates by SDS-PAGE (full antibody list detailed in Table S2, Supplementary Material). Immunoblot quantification conducted with Licor Image Studio software.

### Plasmids

Full length FH-*AGO2* constructs were obtained from Addgene (pIRESneo-FLAG/HA-AGO2 10,822, PI: Thomas Tuschl). *AGO2*Y^393F^ mutant construct was generated using the QuikChange II XL site-directed mutagenesis kit (Agilent) from the FH-*AGO2* plasmid described above using the primers hAGO2_Y393F_Fwd 5“*AAATTCACGGACGAATGGATCTGTGTTGAAACTTGCAC*3’ and hAGO2_Y393F_Rev 5’*GTGCAAGTTTCAACACAGATCCATTCGTCCGTGAATTT*3.” DNA sequences were confirmed using Sanger sequencing at the University of Michigan Sequencing Core.

### Proximity ligation assay

U2OS, T24, and Mel-Juso cell lines were cultured on 8-well chamber slides and serum starved overnight. After indicated treatment/stimulation, cells were fixed with 4% paraformaldehyde, permeabilized using 0.1% Tween. Subsequent Proximity ligation assay (PLA) staining was performed as per the protocol provided by the manufacturer (DUOlink kit, Millipore/Sigma). Antibodies were validated for use previously (11). PLA was performed using RAS10 or AGO2 antibodies either alone or in combination and imaged using a Nikon A1B confocal microscope.

### shRNA viral transduction and *AGO2* knockdown assays

T24, Kasumi-2, Mel-Juso, SK-MEL-2, H1299, HeLa, and A375 cells were treated with two independent shRNAs in viral vectors (validated Mission shRNA lentiviral plasmids, Sigma) targeting *AGO2* (TRCN0000011203 and TRCN0000007867). Cells were incubated at 37°C in 5% CO_2_ and were selected with puromycin over a period of 5 days.

### PTP1B inhibition and siRNA transfection

The Screen-Well Phosphatase Inhibitor Library (Enzolifesiences; BML-2834) was tested against NIH-3T3 *Ago2*^−/−^ (9) at 10 mM concentrations dissolved in DMSO and treated for 2 hours. For hydrogen peroxide treatment, cells were treated in 4 mM H_2_O_2_ for 4 minutes.

### Cellular proliferation assays

Following puromycin selection, *AGO2* knockdown stable cell lines were measured for proliferation through IncuCyte. Approximately 25,000 cells were seeded in triplicate on 24-well plates and measured over a 4- to 5-day period. Confluence rate and standard deviation between replicates were measured on IncuCyte and calculated via Incucyte Zoom software. Kasumi-2 leukemia cell lines were manually counted via hemocytometer (13) in triplicate experiments, and results were analyzed in GraphPad Prism 8 for statistical significance.

### β-galactosidase senescence assay

Following puromycin selection, *AGO2* knockdown stable cell lines were seeded on a 6-well plate. β-galactosidase staining was performed using the Senescence β-Galactosidase Staining Kit #9860 (Cell Signaling) following the established protocol (11).

### Reactive oxygen species assay

Following puromycin selection, *AGO2* knockdown stable cell lines were seeded on a 96-well plate. Reactive oxygen species (ROS) were detected with the ROS-Glo H_2_O_2_ Assay Cat# G8820 (Promega) following the manufacturer's instructions, approximately 24 hours following adherence to dish.

### Molecular dynamics simulations

Coordinates were downloaded for 4W5N (AGO2) (14) from the Protein Data Bank (15). A total of two molecular dynamics simulations (MDS) were run, one with AGO2 Tyr393 phosphorylated and one nonphosphorylated. To make the simulations more feasible, only residues 43 to 406 were used in addition to RNA residues C12 to U21. MOE 2016.08 (16) was used to cap the termini with an acetyl group on the N-terminus and a N-methyl group on the C-terminus. The phosphate was built in manually using MOE 2016.08 (16). Three additional loops were built using MOE's structure preparation tool: residues 150 to 153, Gln246, and residues 273 to 275. Asp, Glu, and His residues were analyzed for appropriate hydrogen bond characteristics and flipped and renamed when necessary. The tleap module of Amber16 (17) was then used with the RNA.OL3 (18) parameters for the RNA and mmff14SB (19) parameters for the protein to neutralize and solvate the system. Parameters for phosphotyrosine were obtained from Homeyer et al. (20). A total of two chloride ions were added to the nonphosphorylated structure to neutralize the system. The solvatebox command in tleap (17) was used to add a 10 Å box of TIP3P water molecules to each system with the closeness parameter set to 0.5.

Each system was minimized using the sander module of amber16 with 250 steps of steepest decent followed by 4,750 steps of conjugate gradient, keeping the protein and RNA fixed. All atoms were then minimized using sander for 250 steps of steepest decent followed by 2,250 steps of conjugate gradient. The temperature was then gradually ramped to 300 K in three steps of 20 ps and one 20 ps step at 300 K keeping the protein and RNA constrained with a 10 kcal/mol·Å^2^ weight. The restrained dynamics were run at 300 K for an additional 250 ps. Equilibration was performed by gradually reducing the weighting on the protein backbone and RNA in two 20 ps runs followed by one 60 ps run with a restraint weight of 0.1 kcal/mol·Å^2^. Completely unrestrained constant pressure and temperature dynamics were then run for 1.4 ns to allow equilibration. Unrestrained constant pressure and temperature molecular dynamics were run for 1.755 µs for the production run using pmemd.cuda (21, 22) on GeForce GTX1080 GPUs. SHAKE was used in all dynamics runs to constrain bonds to hydrogens. A 2-fs timestep was used for all temperature ramping, equilibration, and production runs.

Analysis: The “rmsd” command from the cpptraj module of Amber16 was used to align snapshots from every 500 ps of the production simulation to the starting structure using the Cα atoms. After alignment RMSDs were calculated using the “rmsd” command with the “nofit” option. All snapshots from the molecular dynamics production run were used to calculate correlated motions with the “matrix” command in cpptraj. The correlations were then plotted using the Contour graphing option in JMP13 (23).

## Results

### Identification of the endogenous interaction of AGO2 with HRAS and NRAS

**Fig. 1.**
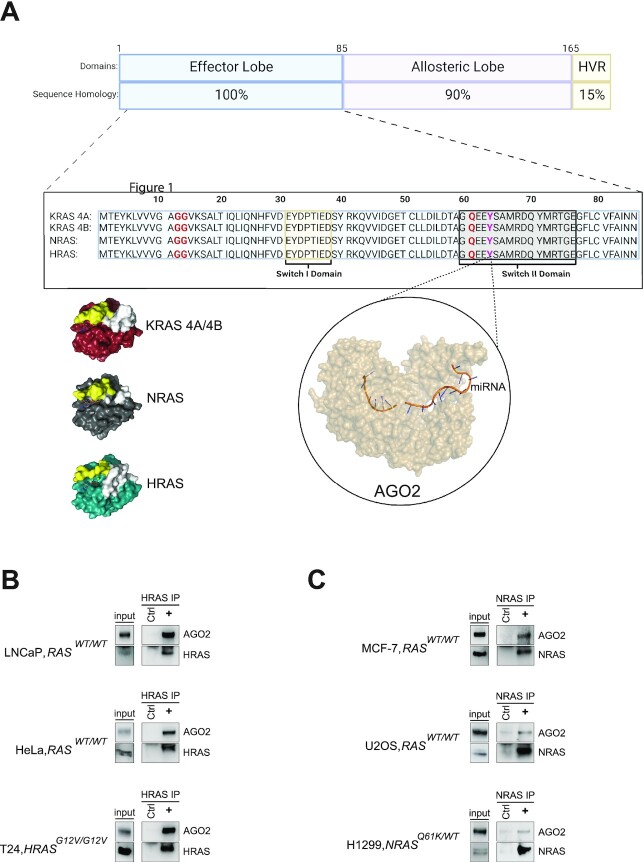
AGO2 interacts with all major isoforms of RAS through their Switch II domains. (A) RAS isoform protein structures and aa homology demonstrate 100% aa conservation at the Switch I domain (highlighted yellow) and Switch II domain (highlighted gray). Commonly mutated aas (G12, G13, and Q61) are highlighted in red. The Switch II domain (residue Y64; highlighted in pink) has previously been identified as the AGO2 binding site on KRAS [9]. Protein structures were generated using Pymol software. Image generated with BioRender.com. HVR, hypervariable region. (B) and (C) Co-IP of endogenous AGO2 with RAS from a panel of benign and cancer cell lines with differing expression of WT or mutant HRAS (B) and NRAS (C).

Our previous work identified a novel interaction between KRAS and AGO2 in both WT and mutant *KRAS* cell lines across multiple cell lineages and cancers via coimmunoprecipitation (Co-IP) followed by mass spectrometry (co-IP MS) (9). Importantly, AGO2 was found to directly interact with the Switch II domain of KRAS through the Y64 residue (9, 10). While nearly 85% of *RAS*-driven cancers are *KRAS* mutants (6), HRAS and NRAS share approximately 82% to 90% of the amino acid (aa) sequence with KRAS (24), with the majority of variance occurring within the C-terminal region (Fig. 1A). This hyperviarable region within the C-terminus of RAS accounts for differences in post-translational modifications between the isoforms (25), ultimately leading to varying membrane trafficking and signaling between them (26).

Considering the sequence homology between RAS isoforms is 100% identical within the Switch II region, we asked whether the interaction between AGO2 and HRAS or NRAS could be detected endogenously in human cancer cell lines. Using a pan-RAS antibody specific to the Switch I domain of RAS (RAS10 (27)), we performed co-IPs across multiple cell lines from diverse cancer types. Interaction with AGO2 was detected in all cell lines expressing either HRAS or NRAS, regardless of mutation status or cell lineage (Fig. 1B and C). Together, these results demonstrate that the AGO2–RAS interaction is consistent across all RAS isoforms and is independent of RAS mutation status.

### EGFR activation disrupts WT RAS–AGO2 but not mutant RAS–AGO2 interaction

**Fig. 2.**
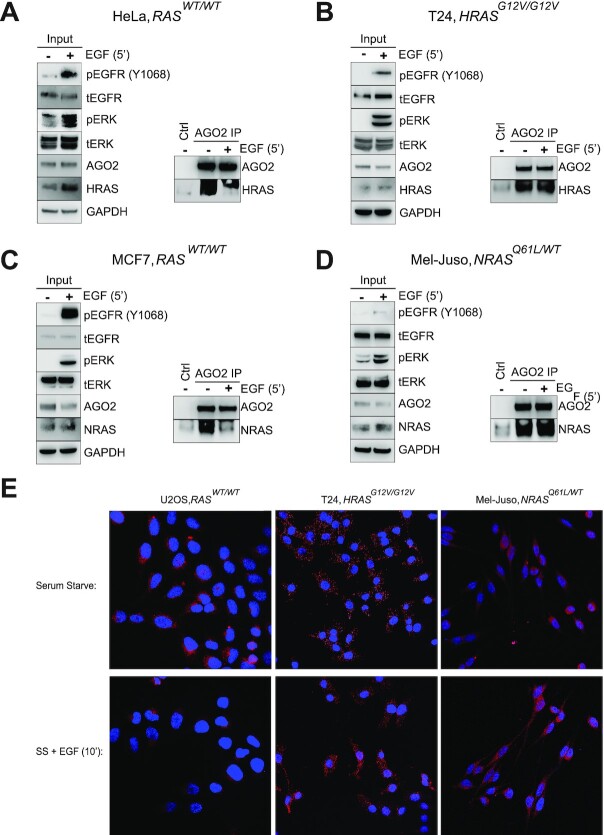
EGF stimulation disrupts WT HRAS–AGO2 and NRAS–AGO2 interaction while mutant RAS–AGO2 interaction is recalcitrant to EGFR activation. (A) IP of endogenous AGO2 upon EGF stimulation (5’) in HeLa cell line expressing WT HRAS followed by immunoblot analysis of HRAS–AGO2 interaction. Ctrl lane on IP represents matched isotype control. (B) Co-IP of endogenous AGO2 following EGF stimulation (5’) in T24 cancer cells harboring *HRAS^G12V/G12V^* mutations, followed by immunoblot analysis of HRAS. Ctrl lane on IP represents matched isotype control. (C) IP of AGO2 following EGF stimulation (5’) in MCF7 cell line expressing WT NRAS with immunoblot analysis of the NRAS–AGO2 interaction. Ctrl lane on IP represents matched isotype control. (D) Co-IP of endogenous AGO2 in Mel-Juso cell with *NRAS^Q61L/WT^* mutation. Ctrl lane on IP represents matched isotype control. For each cell line, MAPK activation and levels of various proteins are shown as input blots. (E) Proximity-ligation assay (PLA) of U2OS (*RAS*-WT), T24 (*HRAS^G12V^*), and Mel-Juso (*NRAS^Q61L^*) following overnight serum starvation followed by EGF stimulation (10’).

Following our demonstration of AGO2’s interaction with both HRAS and NRAS proteins, we next investigated endogenous regulators of this interaction. Upon receptor tyrosine kinase activation, canonical regulators of RAS, such as the GEF and GAP proteins, preferentially bind and determine the GDP/GTP-bound status of WT RAS proteins. These regulators have reduced affinities to oncogenic forms of RAS (28), thereby resulting in a constitutively active, GTP-bound form of mutant RAS. Recent studies demonstrated that EGFR directly binds and phosphorylates AGO2^Y393^ under hypoxic conditions (29). Furthermore, our prior work studying AGO2–KRAS demonstrated regulation of this interaction via EGF stimulation in WT *KRAS* but not mutant *KRAS*-expressing cells (11). These findings prompted us to investigate the effect of EGFR activation on the AGO2–HRAS and NRAS interactions. Using an AGO2-specific antibody (30), we carried out RAS–AGO2 co-IP assays upon stimulation with EGF across a panel of cell lines expressing either *HRAS* or *NRAS*. While basal RAS and AGO2 binding was observed in all of the cell lines, short-term EGF stimulation abolished the WT HRAS–AGO2 interaction in HeLa (Fig. 2A) and LNCaP (Figure S1A, Supplementary Material) cells. Furthermore, short-term EGF stimulation also disrupted the interaction of NRAS–AGO2 in MCF7 (Fig. 2C) and HEK293 (Figure S1C, Supplementary Material) cells expressing WT *NRAS*.

EGFR signaling and cellular trafficking is known to be dysregulated in the presence of mutant *RAS* (31). Considering the connection between EGFR and RAS signaling, we next asked if mutations in *HRAS* could alter growth factor control of the AGO2–HRAS interaction. Interestingly, EGF stimulation in cells harboring oncogenic HRAS, including T24 (*HRAS^G12V/G12V^*; Fig. 2B) (32) and Hs578t (*HRAS^G12D/WT^*; Figure S1B, Supplementary Material) (33), retained binding of endogenous HRAS and AGO2, despite activation of the EGFR/MAPK pathway (Fig. 2B). This was further corroborated in a panel of mutant NRAS cell lines, including Mel-Juso (*NRAS^Q61L/WT^;* Fig. 2D) (34) and H1299 (*NRAS^Q61K/WT^*; Figure S1D, Supplementary Material) (35), which also showed resistance to EGFR regulation of AGO2–NRAS interaction. This suggests that the mutant HRAS–AGO2 and mutant NRAS–AGO2 associations are unaffected by EGF ligand-mediated signaling. Together, these observations represent an intriguing difference in the regulation of the RAS–AGO2 interaction between WT and mutant *HRAS* and *NRAS* that may indicate an important mechanistic difference between the regulation of WT and oncogenic forms of RAS.

To further confirm the disruption of AGO2–RAS interaction and track the localization of these proteins following growth factor activation, we performed PLA on a subset of the cells tested above expressing WT *RAS* (U2OS) (36), mutant *HRAS* (T24), or mutant *NRAS* (Mel-Juso). Our prior studies established that WT AGO2–KRAS was disrupted upon EGF ligand stimulation via IP immunoblot and PLA (11). This was confirmed here via PLA in the U2OS (WT *RAS*) cell line that demonstrated a remarkable loss of AGO2–RAS colocalization following EGF stimulation (Fig. 2E). Conversely, both mutant *HRAS* (T24) and mutant *NRAS* (Mel-Juso) cells demonstrated continued interaction via PLA of AGO2–RAS despite EGFR activation (Fig. 2E). These results corroborate our co-IP analysis and confirm that the mutant HRAS and NRAS–AGO2 interactions are resistant to EGF stimulation.

### Phosphorylation of AGO2 at Y393 leads to conformational change within the RAS binding site

**Fig. 3.**
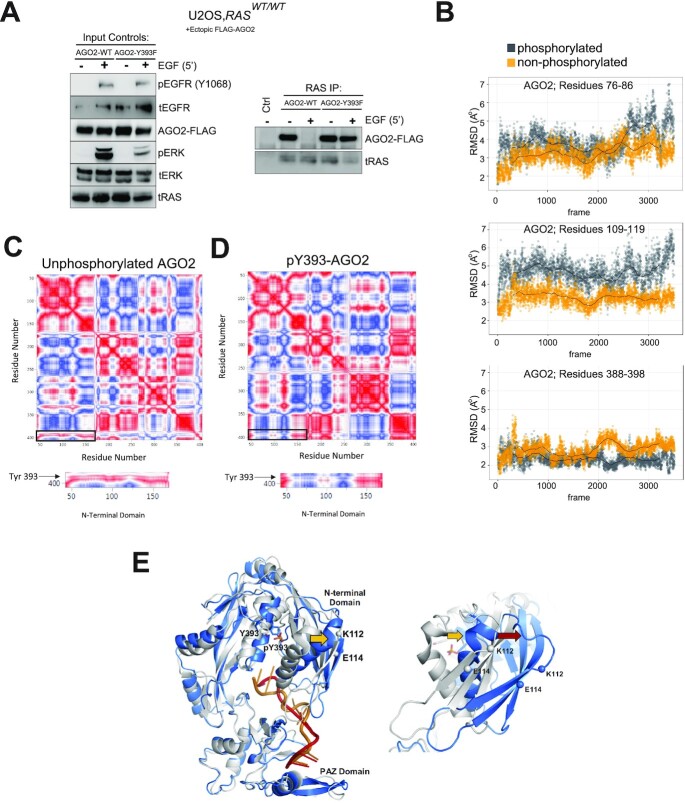
Interaction of AGO2 with all three RAS isoforms is regulated via EGFR pY1068-mediated phosphorylation of Tyrosine 393, resulting in AGO2 protein conformational change. (A) EGF stimulation and total RAS co-IP analysis in U2OS (*RAS^WT/WT^*) cells expressing FLAG-tagged *AGO2* (WT and Y393F). MAPK activation and levels of exogenous FLAG-tagged AGO2 proteins are shown as input blots on left of RAS-IP. Ctrl lane on IP represents matched isotype control. (B) MDS of nonphosphorylated (Y393) and phosphorylated (pY393) AGO2. Root mean square deviation (RMSD) plots of Y393 and pY393 at three different aa stretches (76 to 86, 109 to 119, and 388 to 398 aa), showing average Cα movement of these regions upon phosphorylation. Black lines in plots indicate average movement under each condition. (C) and (D) Correlated motion of Cα atoms from 1.7 μs of MDS for Y393 (C) and pY393 AGO2 (D), respectively. Red = 1 (perfectly correlated), white = 0 (uncorrelated), and blue = −1 (anticorrelated). Below each plot are zoomed images of the N-terminal domain of AGO2. Linear regression analysis of movement differences between nonphosphorylated (Y393) and pY393 AGO2. (E) Overlay of nonphosphorylated (gray) and pY393 (blue) AGO2 structures at the end of simulation. Zoomed-in image of the overlaid N-terminal domain and the stretch involved in RAS binding (109 to 119 aa). Gold arrows represent repulsion of the α-helix in response to phosphorylation, while the red arrow shows movement of the RAS binding site. Processed miRNA is shown in orange for nonphosphorylated and red for pY393 structures. Individual domains (N-terminal-domain and PAZ domain) are labeled.

Previous studies have identified multiple phosphorylation sites in AGO2, including Y529, S387, and Y393 (37), that have been mechanistically associated with AGO2 miRNA binding (38), localization (39), and interactions with RISC members (40), respectively. Phosphorylation at tyrosine 393 downstream of EGFR activation has been described (29), and our prior work demonstrated a role for EGFR phosphorylation at Y393 for disruption of the AGO2–KRAS interaction under multiple conditions (11). To further this observation, we tested the ability of a phosphorylation-deficient AGO2^Y393F^ mutant to bind all three isoforms of RAS under different conditions. In U2OS cells (*RAS*^WT^) (36), EGF stimulation led to dissociation of ectopically overexpressed Flag-AGO2^WT^ from RAS, but the Flag-AGO2^Y393F^ mutant was recalcitrant to EGFR activation, remaining bound to RAS (Fig. 3A). These data confirm that the WT RAS–AGO2 interaction is sensitive to disruption via EGFR-mediated phosphorylation of AGO2^Y393^. Prior studies have suggested that EGFR directly interacts with and phosphorylates AGO2 under multiple conditions including EGF ligand activation and hypoxia; however, EGFR–AGO2 interaction is heavily favored under hypoxic conditions (29). Recently, other intermediate kinases such as c-Src have been identified to phosphorylate AGO2 at multiple tyrosine residues, including Y393 (41). It is, therefore, possible that AGO2 is phosphorylated by multiple kinases downstream of EGFR activation in addition to direct EGFR–AGO2 mediated phosphorylation.

To further probe the effect of AGO2^Y393^ phosphorylation on the AGO2 N-terminal domain, we performed MDS of nonphosphorylated and phosphorylated AGO2 at Y393. Our prior characterization of the AGO2–KRAS interaction identified the K112 and E114 residues of AGO2 as critical for RAS binding at the N-terminus of AGO2. Root mean square deviation (RMSD) overlay plots (42) showed rapid and significant movement in the RAS binding β-sheet stretch spanning aas 109 to 119 (Fig. 3B) when compared to a stretch spanning aas 76 to 86 and the region spanning Y393. In particular, K112 and E114 residues, which are predicted to make contacts (9) in the Switch II domain of RAS, showed significantly greater movement compared to R110 and Y393 (Figure S2, Supplementary Material). In addition, anticorrelated movement of the RAS binding site upon Y393 phosphorylation was observed (Fig. 3C and D). These architectural changes in the N-terminal domain were evident in the structure at the end of simulation (Fig. 3E), and these results suggest that AGO2^Y393^ phosphorylation leads to a conformational sift in the overall protein structure of AGO2’s N-terminus leading to disruption of the RAS binding site at K112 and E114. We propose that the changes observed in this simulation study lead to decreased compatibility of phospho-AGO2^Y393^ with all three RAS isoforms, in addition to the previously described disruption of AGO2–DICER (29). The specific molecular basis of this interaction awaits high resolution structural determination of AGO2 and its complexes with RAS variants.

### AGO2 interaction is essential for mutant *HRAS* and *NRAS*-driven cell proliferation

**Fig. 4.**
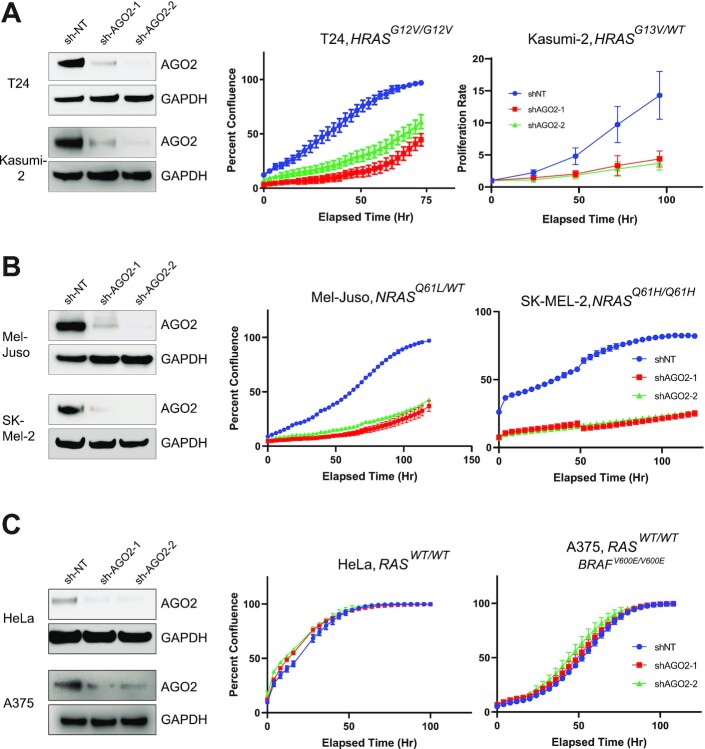
*AGO2* knockdown inhibits proliferation in mutant *HRAS* and *NRAS* but not WT *RAS* cell lines. (A) Immunoblot confirmation of *AGO2* knockdown following stable transduction of shRNA transcripts and matched cell proliferation over time in *HRAS^G12V/G12V^* mutant T24 (transitional cell carcinoma) and *HRAS^G13V/WT^* mutant Kasumi-2 (acute lymphocytic leukemia) cell lines. (B) Matched immunoblot and cell proliferation for *NRAS^Q61L/WT^* mutant Mel-Juso (melanoma) and *NRAS^Q61H/Q61H^* SK-MEL-2 (melanoma) cell lines. (C) WT *RAS* cell lines HeLa (cervical adenocarcinoma) and A375 (melanoma; *BRAF^V600E/V600E^*) with matched cell growth and immunoblot confirmation of AGO2 loss.

After assessing the endogenous regulators of the AGO2–RAS interaction, we next asked if AGO2 played a functional role in promoting mutant *HRAS* and *NRAS* cancers. Using two independent shRNAs targeting *AGO2*, we generated stable transduced cell lines in a variety of mutant *HRAS* and *NRAS*-driven cell lines. As expected, knockdown of *AGO2* led to a profound reduction in cell proliferation in a mutant *HRAS^G12V/G12V^-*driven urinary bladder carcinoma cell line (T24) (32) and a *HRAS^G13V/WT^-*driven acute lymphocytic leukemia cell line (Kasumi-2) (43) compared to a matched nontargeting control shRNA (Fig. 4A; Figure S3, Supplementary Material). Additionally, two mutant *NRAS-*driven melanoma cell lines (Mel-Juso; *NRAS^Q61L/WT^*, and SK-MEL-2; *NRAS^Q61H/Q61H^*) (34, 44) demonstrated marked growth reduction following loss of *AGO2* (Fig. 4B; Figure S3, Supplementary Material). This reduced proliferation potential suggests that AGO2 acts to promote and maintain oncogenic HRAS and NRAS proliferation in mutant *RAS*- driven cells.

Our previous work on the interaction of AGO2 and KRAS demonstrated that *KRAS-*independent cell lines were resistant to loss of *AGO2* (9). Knockdown of *AGO2* expression in the WT *RAS* cell line, HeLa, did not alter cell proliferation (Fig. 4C; Figure S3, Supplementary Material). Interestingly, this was also seen in the melanoma cell line, A375 (45), that harbors the *BRAF^V600E/V600E^* mutation exhibiting a constitutively activated ERK/MAPK pathway, independent of *RAS* mutation (Fig. 4C; Figure S3, Supplementary Material). Furthermore, mutant *Nras^G12D^-*driven cellular transformation of NIH-3T3 cells was potentiated by overexpression of *Ago2*, leading to additional foci formation (Figure S4, Supplementary Material); however, similar to our observations in mutant *BRAF* cell lines, *BRAF^V600E^-*driven transformation was unaffected by overexpression of *AGO2* (9). These results demonstrate that AGO2’s role in mutant *RAS* cells is not recapitulated in constitutive MAPK-driven cancers, further supporting a requirement for the AGO2–RAS interaction in these cells.

### Loss of *AGO2* promotes senescence in mutant *HRAS* and *NRAS*-driven cells through induction of senescence effectors

**Fig. 5.**
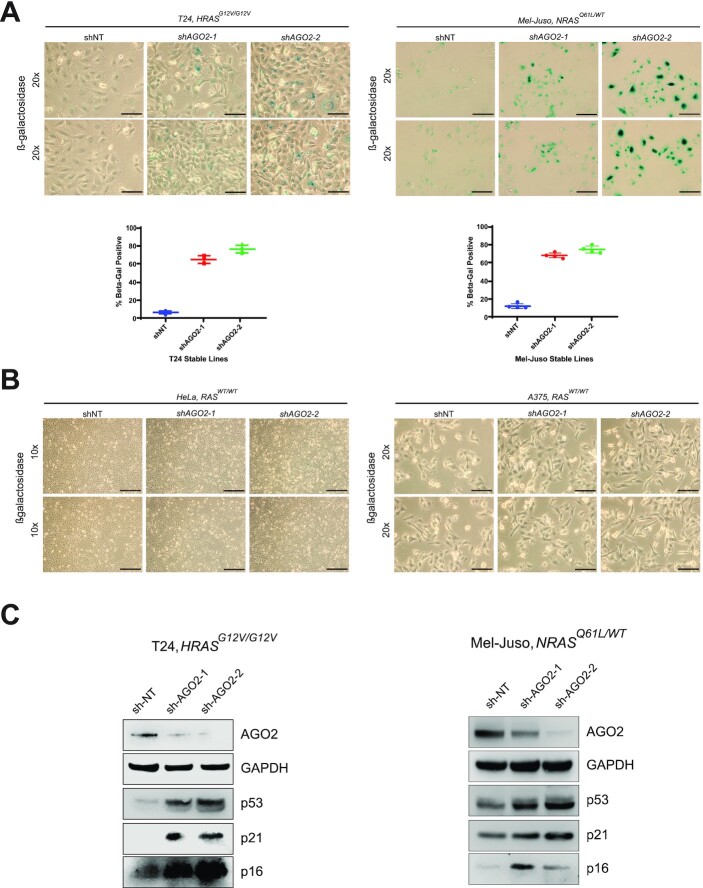
Loss of *AGO2* induces senescence in mutant *HRAS* and *NRAS* cell lines. (A) Representative images following β-galactosidase staining of T24 (*HRAS^G12V/G12V^*) and Mel-Juso (*NRAS^Q61L/WT^*) from stably transduced *AGO2* knockdown cell lines (images at 20x). Matched scatter plot showing % β-galactosidase staining in an average of five images from each condition. (B) β-galactosidase staining of *WT* RAS cell lines following stable knockdown of *AGO2* (HeLa, 10x; A375, 20x). (C) Immunoblot blot analysis of senescence markers (p53, p21, and p16) in stable *AGO2* knockdown T24 (*HRAS^G12V/G12V^*) and Mel-Juso (*NRAS^Q61L/WT^*) cells.

*RAS* genes play a central role in the regulation of the cell cycle through mitogenic MAPK signaling, promoting cell proliferation, and survival (46). In order to better understand the effects of *AGO2* loss on mutant *RAS* cells, we next asked if decreased cell proliferation was due to an induction of cellular senescence, as observed in our prior work in mutant *KRAS* PDAC (11). Following stable *AGO2* knockdown, we performed β-galactosidase staining (47) in the cell line panel described above. β-galactosidase staining is considered a strong biomarker for cellular senescence associated with the build-up in lysosomes (48). Both mutant *HRAS* (T24) and mutant *NRAS* (Mel-Juso)-driven cell lines displayed a significant increase in the number of β-galactosidase-positive cells (Fig. 5A). Furthermore, cells displayed morphologic changes consistent with cellular senescence such as increased size, flat appearance, and enlarged nuclei (49) (Figure S5, Supplementary Material). These results suggest that the loss of *AGO2* led to the induction of senescence in these cells. Additionally, loss of *AGO2* in cell lines expressing WT *RAS* (HeLa and A375) did not lead to an increased level of β-galactosidase staining (Fig. 5B), consistent with continued proliferation following *AGO2* knockdown.

Senescence is regulated via multiple pathways within cells, converging, in part, on activation of tumor suppressor pathways p53/p21 and p16 (50). Cellular senescence has been connected to multiple forms of stress including DNA damage (51), oncogene-induced senescence (OIS) (52), replicative senescence (53), and others. While senescence is controlled by a complex set of signaling events and pathways, induction of p21 and p16 proteins work to inhibit progression through the cell cycle via inhibition of cyclin-dependent kinase phosphorylation of Retinoblastoma (Rb) proteins (50). Activation of Rb via hypophosphorylation leads to cell cycle arrest in G1 phase, inducing cellular senescence (54). Mutations in *RAS* genes are well-known to drive cell cycle arrest in a process known as OIS when introduced to primary fibroblast cells (52). Considering the observed increase in β-galactosidase staining following *AGO2* loss, we asked whether these known senescence pathways were activated in mutant *HRAS* and *NRAS*-driven cell lines. Immunoblot analysis displayed increased expression of p53, p21, and p16 proteins in *AGO2* knockdown cells compared to their nontargeting controls (Fig. 5C; Figure S6, Supplementary Material). The p53/21 and p16 pathways are known to be upregulated following *HRAS^G12V^* overexpression in primary rodent fibroblast cells (52), suggesting that AGO2 may play a role in suppressing OIS in transformed cells. Together, our results demonstrate that loss of *AGO2* is sufficient to induce senescence and arrest cell proliferation via an induction of p53/p21 and p16 senescence effector pathways.

Since mutations or loss of the *TP53* gene leads to evasion of senescence and other tumor suppressor functions in the progression of multiple tumor types (55–57), we asked if *TP53* loss could overcome the observed necessity for *AGO2* in mutant *NRAS*-driven cancers. We selected a NSCLC mutant *NRAS* cell line, H1299 (58), that is *TP53* null. Whereas knockdown of *AGO2* was sufficient to decrease proliferation and induce senescence in our previously tested cell lines, H1299 cells did not demonstrate a sensitivity to *AGO2* loss, maintaining normal growth and negative β-galactosidase staining (Figure S7, Supplementary Material) despite expression of mutant *NRAS*. These findings suggest that *AGO2* is a requirement for mutant *HRAS* and *NRAS*-driven tumor growth that can be circumvented through the loss of *TP53* expression in these cells.

### Loss of *AGO2* promotes inactivation of PTP1B and EGFR activation in a feed forward loop

**Fig. 6.**
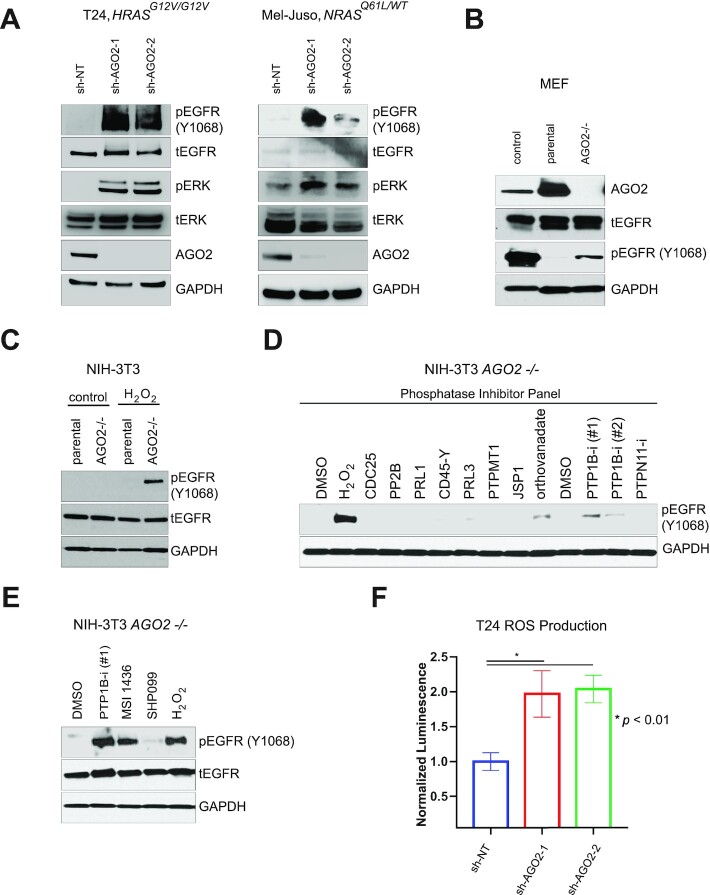
Loss of *AGO2* induces a feedforward loop via ROS-mediated inhibition of PTP1B leading to increased EGFR–RAS–ERK signaling. (A) Immunoblot blot analysis of pERK and pEGFR (Y1068) induction with stable *AGO2* knockdown in T24 (*HRAS^G12V/G12V^*) and Mel-Juso (*NRAS^Q61L/WT^*) cells. (B) Immunoblot analysis of various EGFR tyrosine phosphorylation residues in *Ago2* -/- MEF cells compared to parental MEF and A431 lung cancer cells controls. (C) Increased pEGFR (pY1068) upon treatment with hydrogen peroxide for 4 minutes in NIH-3T3 *Ago2*-/- cells. (D) and (E) Immunoblot analysis of pEGFR (Y1068) upon treatment with various phosphatase inhibitors in NIH-3T3 *Ago2*-/- cells. (F) Normalized ROS production in T24 (*HRAS^G12V/G12V^*) sh*AGO2* knockdown cell lines. Error bars show standard error of mean of three replicates and *P*-value was calculated using two-sided t test.

Since EGFR phosphorylates AGO2 and limits its association with WT RAS (Figs 2 and 3), we next explored whether the EGFR and MAPK pathways could be activated following *AGO2* knockdown in our mutant *HRAS* (T24) and *NRAS* (Mel-Juso) cell line models. Following immunoblotting, we observed not only an increase in pEGFR-Y1068 but also increased levels of pERK (Fig. 6A; Figure S8, Supplementary Material). Activation of the EGFR-Y1068 residue is known to recruit RAS activators like GRB2 and SOS1 in human cells (59), accounting for the observed MAPK signaling. We next assessed whether EGFR levels/activity were altered following *Ago2* loss in mouse embryonic fibroblasts (MEFs) expressing WT *Ras*. We observed increased levels of phosphorylated EGFR residue pY1068 in *Ago2^−^^/^^−^* MEF cells (Fig. 6B; Figure S8, Supplementary Material). Importantly, this residue has previously been shown to activate RAS through the MAPK pathway (60) and is corroborated by our prior observation of MAPK activation in *Ago2^−^^/^^−^* MEF cells (11).

Interestingly, we did not initially observe an increase in pEGFR in our NIH-3T3 *Ago2* knockout cells (Fig. 6C; Figure S9, Supplementary Material). Previous studies have observed a deactivation of the protein phosphatase PTP1B via oxidation by ROS in senescent fibroblasts following the overexpression of mutant *HRAS^G12V^* (61). Additionally, loss of PTP1B activity leads to the phosphorylation of AGO2^Y393^, increasing p21 expression in part through inhibition of AGO2 RNAi function, ultimately progressing to senescence. ROS are well-known products of mutant RAS protein mitogenic signaling (62, 63). As EGFR phosphorylation is a known target of PTP1B (64) and other *AGO2* null fibroblasts show activation of pEGFR (Fig. 6B; Figure S8, Supplementary Material), we treated our NIH-3T3 *Ago2* knockout cell lines with H_2_O_2_. Following hydrogen peroxide treatment, there was a strong induction of pEGFR-Y1068 (Fig. 6C; Figure S8, Supplementary Material) in *Ago2* null cells but not in the parental cells expressing *Ago2*. Considering PTP1B is deactivated by ROS, we next asked if targeted inhibition of this phosphatase could induce similar activation of Y1068-EGFR in vitro. Using a phosphatase inhibitor panel, we saw a large induction of pEGFR-Y1068 in NIH-3T3 *Ago2*^−/−^ cells treated with inhibitors known to target PTP1B (Fig. 6D and E; Figure S8, Supplementary Material) including MSI-1436 (65) and orthovanadate (66).

Given the potential connection between PTP1B inactivation and EGFR activation in cells deficient in AGO2, we next asked if *AGO2* loss was associated with production of ROS in our mutant *HRAS* and mutant *NRAS* models. Importantly, prior studies have implicated ROS in the promotion of senescence through multiple pathways including mutant *RAS* activation (62). We observed a strong increase in production of ROS following *AGO2* knockdown in both *HRAS* (Fig. 6F) and *NRAS* (Figure S10, Supplementary Material) mutant cell lines. Along with our earlier observations that *AGO2* knockdown induced OIS in mutant *HRAS* and *NRAS* cell lines (Fig. 5), these results suggest an important new feed forward loop in mutant *RAS*-driven cells following the loss of *AGO2* through inhibition of PTP1B regulation of EGFR-mediated signaling.

## Discussion

**Fig. 7.**
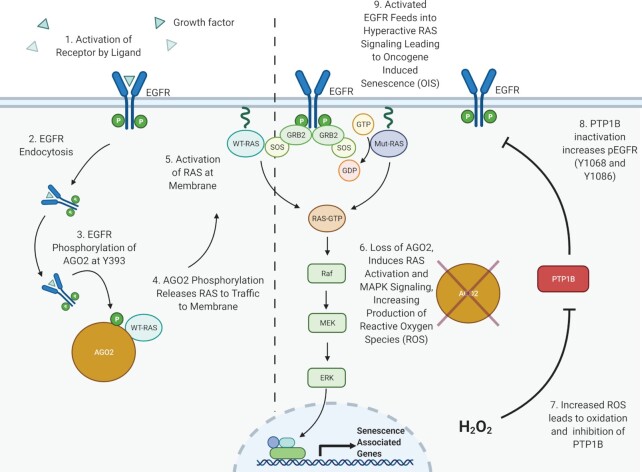
A model for EGFR regulation of AGO2–RAS interaction, and inhibition of PTP1B through *AGO2* loss driving OIS. Dotted line separates WT-RAS (left side) and Mutant-RAS (right side) expressing cells. In normal, WT-RAS (blue) expressing cells (steps 1 to 5; left of dotted line): (1) EGFR is activated by EGF ligand binding, (2) pEGFR is endocytosed and interacts with AGO2, (3) phosphorylation of AGO2 leads to a conformational change, leading to (4) displacement of RAS, and (5) increased activation of RAS at the membrane. In mutant RAS (purple) expressing cells (steps 6 to 9; right of dotted line), the loss of *AGO2* leads to (6) increased production of ROS and increased MAPK signaling, (7) leading to oxidation and inhibition of PTP1B phosphatase activity, (8) inactivation of PTP1B causes an increase in pEGFR (Y1068), which in turn, (9) feeds forward to cause hyperactive RAS signaling, further MAPK activation, and ultimately resulting in oncogene induced senescence. Created with BioRender.com.

Despite years of research, prior attempts to clinically target *RAS* mutations have been largely unsuccessful (67), and efforts to target downstream RAS signaling or subcellular localization have demonstrated limited efficacy (68). Recently, small molecule inhibitors of KRAS^G12C^ point mutations have shown promise in human trials (69, 70); however, no specific inhibitors of mutant NRAS or HRAS have been successfully developed to date (71). With the goal of identifying potentially druggable interactors with mutant RAS proteins, our group recently identified the novel KRAS–AGO2 interaction (9) and described its role in promoting both pancreatic (11) and NSCLC (12). Importantly, this interaction was specific to AGO2 and not the other Argonaute isoforms (9). In parallel to our studies with mutant *KRAS*, we set out to identify a role for AGO2 in mutant *HRAS* and *NRAS-*driven cancers. In this manuscript, we describe the endogenous interaction of AGO2 with both HRAS and NRAS. Furthermore, we describe two novel mechanistic insights into the role of the EGFR–AGO2–RAS signaling axis in promoting mutant *RAS*-driven oncogenesis (Fig. 7).

First, we describe the role of EGFR-mediated phosphorylation of residue Y393 on AGO2, leading to a novel conformational change in the N-terminal domain of AGO2 (Fig. 3). This proposed structural change accounts for the disruption of AGO2–RAS interaction and promotion of RAS activation through increased interaction with RAS effectors like SOS1 (11). Oncogenic HRAS and NRAS, however, are resistant to this disruption downstream of EGF ligand. Our data also show that EGFR-mediated phosphorylation of AGO2^Y393^ disrupts binding of all three isoforms of WT RAS. This event both regulates WT–RAS interaction with AGO2 and inhibits microRNA processing via disruption of AGO2–DICER interaction. While phosphorylation of AGO2^Y393^ itself has not been associated with changes in miRNA binding, increased Y393 phosphorylation was linked to inhibited processing of long-loop miRNA transcripts into mature miRNA (29). Interestingly, our prior work demonstrated that mutant KRAS inhibited AGO2 unwinding of miRNA duplexes (9). These findings suggest that while mutant RAS bound AGO2 is still loaded with miRNA transcripts, this interaction may be resistant to RISC formation and awaits further investigation.

Our MDS analyses suggest that AGO2 phosphorylation at Y393 leads to conformational changes within the RAS binding region (K112 and E114), likely destabilizing the WT RAS binding. While the mutant RAS–AGO2 binding may be recalcitrant to such changes in 3D structure, our prior work has not demonstrated any increased affinity of mutant KRAS–AGO2 complexes compared to WT RAS (10). It is also possible that EGFR is unable to access and phosphorylate AGO2 in mutant *RAS*-expressing cells under normal EGF ligand-driven activation, which may exist in spatially distinct complexes. Furthermore, our observation of resistance to EGFR phosphorylation of AGO2 in the setting of mutant *HRAS* or *NRAS* may be the result of additional dysregulation of protein phosphatases such as PTP1B, leading to promotion of RAS–AGO2 binding. Recent work by other groups has also found that mutant KRAS promotes phosphorylation of AGO2^S387^ through MAPK signaling, leading to alteration of miRNA maturation and exosome sorting (39).

Second, we characterized a novel feed forward pathway in which EGFR–MAPK signaling is activated downstream of *AGO2* knockdown in mutant *HRAS* and *NRAS* cell lines, ultimately leading to the observed OIS phenotype. Activation of MAPK signaling downstream of *AGO2* loss was observed previously in our mutant *KRAS*-driven model of PDAC through an induction of WT RAS–GTP signaling (11). In this study, we observed that increased mitogenic signaling results in elevated ROS production following *AGO2* loss, leading to inactivation of PTP1B and subsequent accumulation of active pEGFR-Y1068. This increased pEGFR feeds forward, activating RAS and downstream MAPK signaling, further promoting OIS in these cells (Fig. 7). Interestingly, AGO2 has previously been linked to OIS driven by *HRAS^G12V^* in fibroblasts. Phosphorylation of AGO2^Y393^ led to increased p21 expression and senescence following the inactivation of protein phosphatase PTP1B (61). Our model suggests that phosphorylation of Y393–AGO2 leads to the disruption of HRAS–AGO2 and NRAS–AGO2 interaction. Additionally, the loss of *AGO2* inhibits mutant *HRAS* and *NRAS* growth in part through induction of senescent pathways like p53 and p16 (Fig. 5). Finally, the loss of *AGO2* leads to the production of ROS causing the inactivation of PTP1B and the induction of a pEGFR–RAS–ERK feed forward loop (Fig. 6). Our previous work in a genetically engineered mouse model of *KRAS*-driven PDAC demonstrated that AGO2 can partially inhibit KRAS interaction with SOS1. GEFs, like SOS1, play an important role in the activation of WT RAS by promoting the release of GDP, ultimately leading to the loading of GTP into RAS (72). Our in vitro data demonstrating this inhibition of KRAS–SOS1 activity was confirmed in vivo where *Ago2* loss led to an increase in RAS–GTP loading in both WT *RAS* MEF cells and PDAC PanIN organoid cell culture (11). This observed EGFR–AGO2–RAS signaling axis suggests that the continued binding of RAS and AGO2 is, in part, required to prevent OIS and can be overcome through loss of *AGO2* expression.

Despite recent progress in targeting KRAS^G12C^ mutations (69), few treatment options remain available to target RAS clinically. Taken together, our study finds a novel role for AGO2 as a regulator of cellular proliferation and senescence in mutant *HRAS* and *NRAS-*driven cancers through an EGFR–AGO2–RAS signaling axis. While further efforts to target this interaction await cocrystalization and structural determination of AGO2 with RAS variants, a greater understanding of the AGO2–RAS interaction in human cancer may inform future clinical targeting of mutant RAS.

## Supplementary Material

pgac084_Supplemental_Files

## Data Availability

All data is included in the manuscript and/or supporting information.

## References

[1] Simanshu DK , Nissley DV, McCormick F. 2017. RAS proteins and their regulators in human disease. Cell. 170(1):17–33. 28666118 10.1016/j.cell.2017.06.009 PMC5555610

[2] Pylayeva-Gupta Y , Grabocka E, Bar-Sagi D. 2011. RAS oncogenes: weaving a tumorigenic web. Nat Rev Cancer. 11(11):761–774. 21993244 10.1038/nrc3106 PMC3632399

[3] Stephen AG et al. 2014. Dragging ras back in the ring. Cancer Cell. 25(3):272–281. 24651010 10.1016/j.ccr.2014.02.017

[4] Cox AD , Der CJ. 2010. Ras history: the saga continues. Small GTPases. 1(1):2–27. 21686117 10.4161/sgtp.1.1.12178 PMC3109476

[5] Kleeff J et al. 2016. Pancreatic cancer. Nat Rev Dis Primers. 2:16022. 27158978 10.1038/nrdp.2016.22

[6] Hobbs G.A. , Der C.J., Rossman KL. 2016. RAS isoforms and mutations in cancer at a glance. J Cell Sci. 129(7):1287–1292. 26985062 10.1242/jcs.182873 PMC4869631

[7] Rajalingam K 2007. Ras oncogenes and their downstream targets. Biochim Biophys Acta. 1773(8):1177–1195. 17428555 10.1016/j.bbamcr.2007.01.012

[8] McCormick F . 2015. KRAS as a therapeutic target. Clin Cancer Res. 21(8):1797–1801. 25878360 10.1158/1078-0432.CCR-14-2662 PMC4407814

[9] Shankar S 2016. KRAS engages AGO2 to enhance cellular transformation. Cell Rep. 14(6):1448–1461. 26854235 10.1016/j.celrep.2016.01.034 PMC4758864

[10] Waninger JJ et al. 2022. Biochemical characterization of the interaction between KRAS and Argonaute 2. Biochem Biophys Rep. 29:101191. 34988297 10.1016/j.bbrep.2021.101191 PMC8695255

[11] Shankar S et al. 2020. An essential role for Argonaute 2 in EGFR-KRAS signaling in pancreatic cancer development. Nat Commun. 11(1):2817. 32499547 10.1038/s41467-020-16309-2 PMC7272436

[12] Tien JC 2021. AGO2 promotes tumor progression in KRAS-driven mouse models of non-small cell lung cancer. Proc Natl Acad Sci USA. 118(20):e2026104118. 33972443 10.1073/pnas.2026104118 PMC8157917

[13] Green MR , Sambrook J. 2019. Estimation of cell number by hemocytometry counting. Cold Spring Harb Protoc. 2019(11):31676576. 10.1101/pdb.prot097980 31676576

[14] Schirle NT , Sheu-Gruttadauria J, MacRae IJ. 2014. Structural basis for microRNA targeting. Science. 346(6209):608–613. 25359968 10.1126/science.1258040 PMC4313529

[15] Berman HM 2000. The protein data bank. Nucleic Acids Res. 28:235–242. 10592235 10.1093/nar/28.1.235 PMC102472

[16] CCG Inc. 2016. MOE 2016.08. Montreal, QC.

[17] Case DA et al. 2017. AMBER 2017. Editor. San Francisco, CA: University of California.

[18] Zgarbova M et al. 2011. Refinement of the cornell et al. nucleic acids force field based on reference quantum chemical calculations of glycosidic torsion profiles. J Chem Theory Comput. 7(9):2886–2902. 21921995 10.1021/ct200162x PMC3171997

[19] Maier JA et al. 2015. ff14SB: improving the accuracy of protein side chain and backbone parameters from ff99SB. J Chem Theory Comput. 11(8):3696–3713. 26574453 10.1021/acs.jctc.5b00255 PMC4821407

[20] Homeyer N et al. 2016. AMBER force-field parameters for phosphorylated amino acids in different protonation states: phosphoserine, phosphothreonine, phosphotyrosine, and phosphohistidine. J Mol Model. 12(3):281–289. 10.1007/s00894-005-0028-4 16240095

[21] Goetz AW et al. 2012. Routine microsecond molecular dynamics simulations with AMBER on GPUs. 1. generalized born. J Chem Theory Comput. 8(5):1542–1555. 22582031 10.1021/ct200909j PMC3348677

[22] Salomon-Ferrer R 2013. Routine microsecond molecular dynamics simulations with AMBER on GPUs. 2. explicit solvent particle mesh ewald. J Chem Theory Comput. 9(9):3878–3888. 26592383 10.1021/ct400314y

[23] Wang L et al. 2016. Transcriptomic characterization of SF3B1 mutation reveals its pleiotropic effects in chronic lymphocytic leukemia. Cancer Cell. 30(5):750–763. 27818134 10.1016/j.ccell.2016.10.005 PMC5127278

[24] Zhou B , Der CJ, Cox AD. 2016. The role of wild type RAS isoforms in cancer. Semin Cell Dev Biol. 58:60–69. 27422332 10.1016/j.semcdb.2016.07.012 PMC5028303

[25] Hancock JF 1989. All ras proteins are polyisoprenylated but only some are palmitoylated. Cell. 57(7):1167–1177. 2661017 10.1016/0092-8674(89)90054-8

[26] Hancock JF . 2003. Ras proteins: different signals from different locations. Nat Rev Mol Cell Biol. 4(5):373–384. 12728271 10.1038/nrm1105

[27] Cheng CM et al. 2011. Compartmentalized ras proteins transform NIH 3T3 cells with different efficiencies. Mol Cell Biol. 31(5):983–997. 21189290 10.1128/MCB.00137-10 PMC3067814

[28] Munoz-Maldonado C , Zimmer Y, Medova M. 2019. A comparative analysis of individual RAS mutations in cancer biology. Front Oncol. 9:1088. 31681616 10.3389/fonc.2019.01088 PMC6813200

[29] Shen J et al. 2013. EGFR modulates microRNA maturation in response to hypoxia through phosphorylation of AGO2. Nature. 497(7449):383–387. 23636329 10.1038/nature12080 PMC3717558

[30] Rudel S et al. 2008. A multifunctional human Argonaute2-specific monoclonal antibody. RNA. 14(6):1244–1253. 18430891 10.1261/rna.973808 PMC2390805

[31] van Houdt WJ et al. 2010. Oncogenic KRAS desensitizes colorectal tumor cells to epidermal growth factor receptor inhibition and activation. Neoplasia. 12(6):443–452. 20563247 10.1593/neo.92088 PMC2887497

[32] Reddy EP et al. 1982. A point mutation is responsible for the acquisition of transforming properties by the T24 human bladder carcinoma oncogene. Nature. 300(5888):149–152. 7133135 10.1038/300149a0

[33] Hollestelle A 2010. Distinct gene mutation profiles among luminal-type and basal-type breast cancer cell lines. Breast Cancer Res Treat. 121(1):53–64. 19593635 10.1007/s10549-009-0460-8

[34] Tsao H 2000. Relative reciprocity of NRAS and PTEN/MMAC1 alterations in cutaneous melanoma cell lines. Cancer Res. 60(7):1800–1804. 10766161

[35] Ohashi K et al. 2013. Characteristics of lung cancers harboring NRAS mutations. Clin Cancer Res. 19(9):2584–2591. 23515407 10.1158/1078-0432.CCR-12-3173 PMC3643999

[36] Ottaviano L et al. 2010. Molecular characterization of commonly used cell lines for bone tumor research: a trans-European EuroBoNet effort. Genes Chromosomes Cancer. 49(1):40–51. 19787792 10.1002/gcc.20717

[37] Lopez-Orozco J et al. 2015. Functional analyses of phosphorylation events in human Argonaute 2. RNA. 21(12):2030–2038. 26443379 10.1261/rna.053207.115 PMC4647457

[38] Rudel S 2011. Phosphorylation of human Argonaute proteins affects small RNA binding. Nucleic Acids Res. 39(6):2330–2343. 21071408 10.1093/nar/gkq1032 PMC3064767

[39] McKenzie AJ et al. 2016. KRAS-MEK signaling controls Ago2 sorting into exosomes. Cell Rep. 15(5):978–987. 27117408 10.1016/j.celrep.2016.03.085 PMC4857875

[40] Jee D , Lai EC. 2014. Alteration of miRNA activity via context-specific modifications of Argonaute proteins. Trends Cell Biol. 24(9):546–553. 24865524 10.1016/j.tcb.2014.04.008 PMC4149831

[41] Liu T et al. 2020. AGO2 phosphorylation by c-Src kinase promotes tumorigenesis. Neoplasia. 22(3):129–141. 31981897 10.1016/j.neo.2019.12.004 PMC6992904

[42] Damm KL , Carlson HA. 2006. Gaussian-weighted RMSD superposition of proteins: a structural comparison for flexible proteins and predicted protein structures. Biophys J. 90(12):4558–4573. 16565070 10.1529/biophysj.105.066654 PMC1471868

[43] Duchartre Y et al. 2017. Effects of CD49d-targeted antisense-oligonucleotide on alpha4 integrin expression and function of acute lymphoblastic leukemia cells: results of in vitro and in vivo studies. PLoS ONE. 12(11):e0187684. 29117236 10.1371/journal.pone.0187684 PMC5678723

[44] Vogel CJ 2015. Cooperative induction of apoptosis in NRAS mutant melanoma by inhibition of MEK and ROCK. Pigment Cell Melanoma Res. 28(3):307–317. 25728708 10.1111/pcmr.12364

[45] Tanami H et al. 2004. Involvement of overexpressed wild-type BRAF in the growth of malignant melanoma cell lines. Oncogene. 23(54):8796–8804. 15467732 10.1038/sj.onc.1208152

[46] Macaluso M et al. 2002. Ras family genes: an interesting link between cell cycle and cancer. J Cell Physiol. 192(2):125–130. 12115718 10.1002/jcp.10109

[47] Dimri GP et al. 1995. A biomarker that identifies senescent human cells in culture and in aging skin in vivo. Proc Natl Acad Sci USA. 92(20):9363–9367. 7568133 10.1073/pnas.92.20.9363 PMC40985

[48] Lee BY et al. 2006. Senescence-associated beta-galactosidase is lysosomal beta-galactosidase. Aging Cell. 5(2):187–195. 16626397 10.1111/j.1474-9726.2006.00199.x

[49] Kuilman T et al. 2010. The essence of senescence. Genes Dev. 24(22):2463–2479. 21078816 10.1101/gad.1971610 PMC2975923

[50] Lee S , Lee JS. 2019. Cellular senescence: a promising strategy for cancer therapy. BMB Rep. 52(1):35–41. 30526771 10.5483/BMBRep.2019.52.1.294 PMC6386234

[51] d'Adda di Fagagna F et al. 2003. A DNA damage checkpoint response in telomere-initiated senescence. Nature. 426(6963):194–198. 14608368 10.1038/nature02118

[52] Serrano M et al. 1997. Oncogenic ras provokes premature cell senescence associated with accumulation of p53 and p16INK4a. Cell. 88(5):593–602. 9054499 10.1016/s0092-8674(00)81902-9

[53] Hayflick L , Moorhead PS. 1961. The serial cultivation of human diploid cell strains. Exp Cell Res. 25:585–621. 13905658 10.1016/0014-4827(61)90192-6

[54] Chicas A et al. 2010. Dissecting the unique role of the retinoblastoma tumor suppressor during cellular senescence. Cancer Cell. 17(4):376–387. 20385362 10.1016/j.ccr.2010.01.023 PMC2889489

[55] Morton JP et al. 2010. Mutant p53 drives metastasis and overcomes growth arrest/senescence in pancreatic cancer. Proc Natl Acad Sci USA. 107(1):246–251. 20018721 10.1073/pnas.0908428107 PMC2806749

[56] Xue W et al. 2007. Senescence and tumour clearance is triggered by p53 restoration in murine liver carcinomas. Nature. 445(7128):656–660. 17251933 10.1038/nature05529 PMC4601097

[57] Symonds H et al. 1994. p53-dependent apoptosis suppresses tumor growth and progression in vivo. Cell. 78(4):703–711. 8069917 10.1016/0092-8674(94)90534-7

[58] Lin DL , Chang C. 1996. p53 is a mediator for radiation-repressed human TR2 orphan receptor expression in MCF-7 cells, a new pathway from tumor suppressor to member of the steroid receptor superfamily. J Biol Chem. 271(25): 14649–14652. 8663350 10.1074/jbc.271.25.14649

[59] Yamaoka T et al. 2011. Specific epidermal growth factor receptor autophosphorylation sites promote mouse colon epithelial cell chemotaxis and restitution. Am J Physiol Gastrointest Liver Physiol. 301(2):G368–G376. 21617115 10.1152/ajpgi.00327.2010 PMC3154598

[60] Lowenstein EJ et al. 1992. The SH2 and SH3 domain-containing protein GRB2 links receptor tyrosine kinases to ras signaling. Cell. 70(3):431–442. 1322798 10.1016/0092-8674(92)90167-b

[61] Yang M et al. 2014. Dephosphorylation of tyrosine 393 in argonaute 2 by protein tyrosine phosphatase 1B regulates gene silencing in oncogenic RAS-induced senescence. Mol Cell. 55(5):782–790. 25175024 10.1016/j.molcel.2014.07.018 PMC4159145

[62] Lee AC et al. 1999. Ras proteins induce senescence by altering the intracellular levels of reactive oxygen species. J Biol Chem. 274(12):7936–7940. 10075689 10.1074/jbc.274.12.7936

[63] Ogrunc M et al. 2014. Oncogene-induced reactive oxygen species fuel hyperproliferation and DNA damage response activation. Cell Death Differ. 21(6):998–1012. 24583638 10.1038/cdd.2014.16 PMC4013514

[64] Ishino Y et al. 2008. Protein tyrosine phosphatase-1B (PTP1B) helps regulate EGF-induced stimulation of S-phase entry in human corneal endothelial cells. Mol Vis. 14:61–70. 18253097 PMC2263008

[65] Smith AM et al. 2017. The protein tyrosine phosphatase 1B inhibitor MSI-1436 stimulates regeneration of heart and multiple other tissues. NPJ Regen Med. 2:4. 29302341 10.1038/s41536-017-0008-1 PMC5677970

[66] Kumar S et al. 2019. Attenuation of hyperhomocysteinemia induced vascular dementia by sodium orthovanadate perhaps via PTP1B: pertinent downstream outcomes. Behav Brain Res. 364:29–40. 30721761 10.1016/j.bbr.2019.01.039

[67] Cox AD et al. 2014. Drugging the undruggable RAS: mission possible?. Nat Rev Drug Discov. 13(11):828–851. 25323927 10.1038/nrd4389 PMC4355017

[68] Chen K et al. 2021. Emerging strategies to target RAS signaling in human cancer therapy. J Hematol Oncol. 14(1):116. 34301278 10.1186/s13045-021-01127-w PMC8299671

[69] Ostrem JM et al. 2013. K-Ras(G12C) inhibitors allosterically control GTP affinity and effector interactions. Nature. 503(7477):548–551. 24256730 10.1038/nature12796 PMC4274051

[70] Skoulidis F et al. 2021. Sotorasib for lung cancers with KRAS p.G12C mutation. N Engl J Med. 384(25):2371–2381. 34096690 10.1056/NEJMoa2103695 PMC9116274

[71] Moore AR et al. 2020. RAS-targeted therapies: is the undruggable drugged?. Nat Rev Drug Discov. 19(8):533–552. 32528145 10.1038/s41573-020-0068-6 PMC7809886

[72] Vetter IR , Wittinghofer A. 2001. The guanine nucleotide-binding switch in three dimensions. Science. 294(5545):1299–1304. 11701921 10.1126/science.1062023

