# Supplementary material for: Argonaute 2 modulates EGFR–RAS signaling to promote mutant HRAS and NRAS-driven malignancies
Source: PNAS Nexus. 2022 Jul 28;1(3):pgac084. doi: 10.1093/pnasnexus/pgac084 (PMC9338400; doi:10.1093/pnasnexus/pgac084)
Supplement: pgac084_Supplemental_Files [file pgac084_supplemental_files.zip › PNASNEXUS-PNASNEXUS-2022-00105-s01.pdf]

Table S1: Cell lines used in study

Number	Cell Line	Source	Tissue	Type	*RAS* or *AGO2* Status
1	LNCaP	Human	Prostate	Cancer	*RAS-WT*
2	HeLa	Human	Cervix	Cancer	*RAS-WT*
3	HEK293	Human	Embryonic Kidney	Benign	*RAS-WT*
4	MCF-7	Human	Breast	Cancer	*RAS-WT*
5	U2OS	Human	Bone	Cancer	*RAS-WT*
6	A375	Human	Melanoma	Cancer	*RAS-WT*
7	Hs578t	Human	Breast	Cancer	*HRAS-G12D*
8	T24	Human	Bladder	Cancer	*HRAS-G12V*
9	Kasumi-2	Human	ALL	Cancer	*HRAS-G13V*
10	Mel-Juso	Human	Melanoma	Cancer	*NRAS-Q61L*
11	SK-MEL-2	Human	Melanoma	Cancer	*NRAS-Q61H*
12	H1299	Human	Lung	Cancer	*NRAS-Q61K*
13	NIH-3T3, Parental	Mouse	Embryonic Fibroblast	Benign	*RAS-WT; AGO2 WT*
14	NIH-3T3, *AGO2 -/-*	Mouse	Embryonic Fibroblast	Benign	*RAS-WT; AGO2 -/-*
15	MEF, Parental	Mouse	Embryonic Fibroblast	Benign	*RAS-WT; AGO2 WT*
16	MEF, AGO2 -/-	Mouse	Embryonic Fibroblast	Benign	*RAS-WT; AGO2 -/-*

Table S2: Antibodies used in study

#	Antibody	Vendor	Catalog Number	Application	Specificity
1	Anti-Ras clone 10 (RAS10)	Millipore	05-516	IP, IB, PLA	Hu and Ms
2	HRAS	Proteintech	18295–1-AP	IB	Hu and Ms
3	NRAS	Proteintech	10724–1-AP	IB	Hu and Ms
4	NRAS	Santa Cruz	sc-31	IB	Hu and Ms
5	AGO2, 11A9	Sigma	SAB4200085	IP, IB	Hu
6	AGO2, EIF2C2	Sino Biologicals	11079-T36	IB, PLA	Hu and Ms
7	Anti-EGFR (phospho Y1086)	Cell Signaling	2220S	IB	Hu and Ms
8	Anti-EGFR (phospho Y1068)	Cell Signaling	2234S	IB	Hu and Ms
9	Anti-EGFR (A-10)	Santa Cruz	sc-373746	IB	Hu and Ms
10	Anti-EGFR	Millipore	06-847	IB	Hu
11	Phospho-p44/42 MAPK (Erk 1/2)	Cell Signaling	4376	IB	Hu and Ms
12	Total p44/42 MAPK (Erk 1/2)	Cell Signaling	9102	IB	Hu
13	GAPDH-HRP	Cell Signaling	3683	IB	Hu and Ms
14	Anti-FLAG antibody	Sigma	F7425-0.2MG	IB	Hu and Ms
15	Anti-p53	Sigma	P8999-200UL	IB	Hu
16	p21 Waf1/Cip1 (12D1)	Cell Signaling	2947S	IB	Hu
17	p16 INK4A (D7C1M)	Cell Signaling	80772S	IB	Hu
18	Normal Mouse IgG	Santa Cruz	sc-2025	Isotype Control IP	Ms
19	Normal Rat IgG	Abcam	ab18450	Isotype Control IP	Rat

IP: Immunoprecipitation; IB: Immunoblot; Hu: Human; Ms: Mouse; PLA: Proximity Ligation Assay

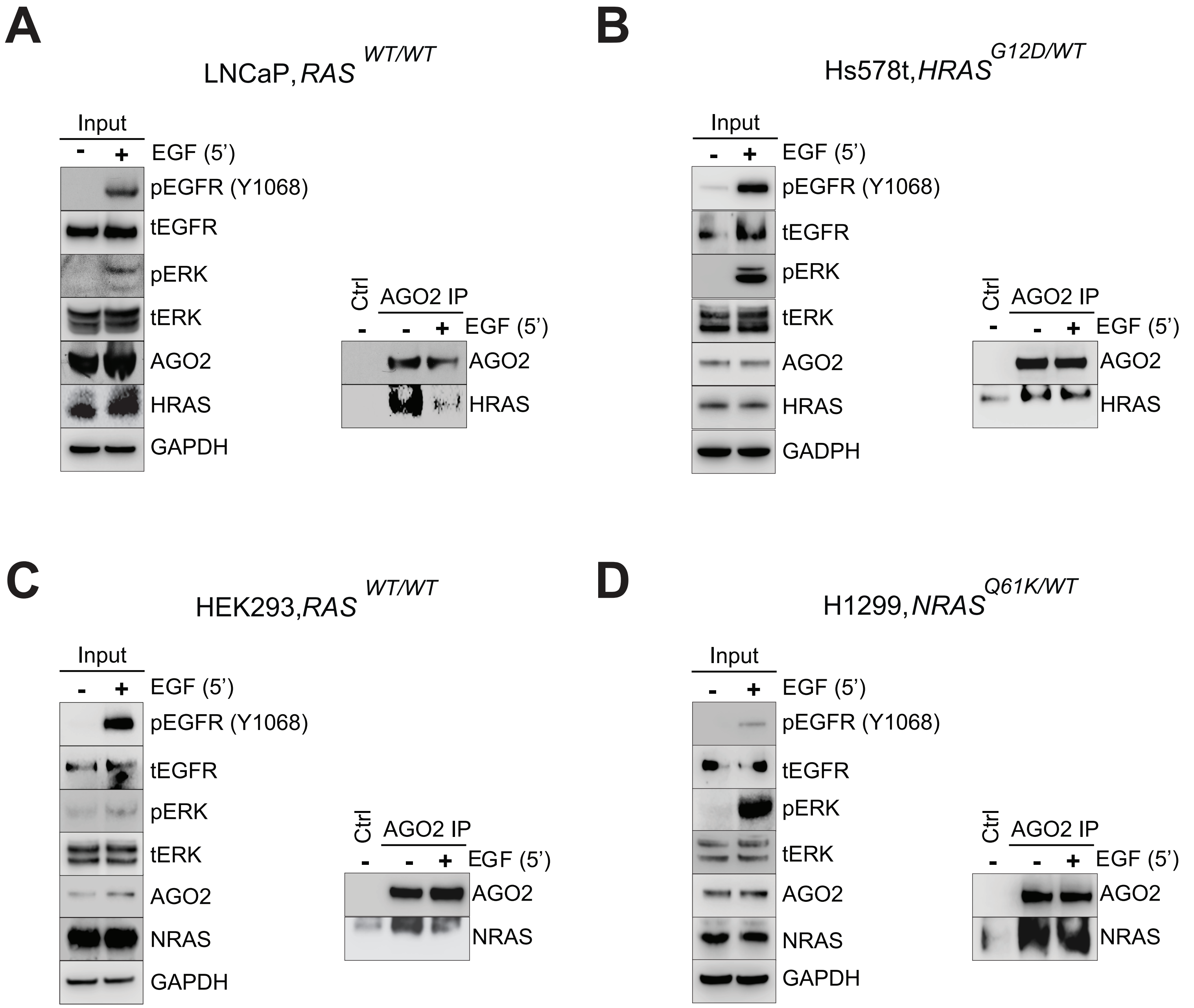

Figure S1. EGF stimulation disrupts WT HRAS-AGO2 and NRAS-AGO2 interaction while mutant RAS-AGO2 interaction is recalcitrant to EGFR activation across multiple cell lines and lineages. (**A**) IP of endogenous AGO2 upon EGF stimulation (5’) in LNCaP cell line expressing WT HRAS followed by immunoblot analysis of HRAS-AGO2 interaction. Ctrl lane on IP represents matched isotype control. (**B**) Co-IP of endogenous AGO2 following EGF stimulation (5’) in Hs578t cancer cells harboring *HRAS*$^{G12D/WT}$ mutation, followed by immunoblot analysis of HRAS. Ctrl lane on IP represents matched isotype control. (**C**) Immunoprecipitation of AGO2 following EGF stimulation (5’) in Hek293 cell line expressing WT NRAS with immunoblot analysis of the NRAS-AGO2 interaction. Ctrl lane on IP represents matched isotype control. (**D**) Co-IP of endogenous AGO2 in H1299 cell with *NRAS*$^{Q61K/WT}$ mutation. Ctrl lane on IP represents matched isotype control. For each cell line, MAPK activation and levels of various proteins are shown as input blots.

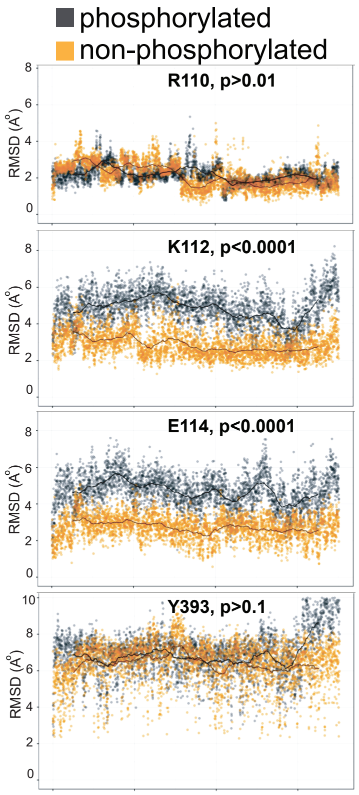

Figure S2. Molecular dynamics simulation of various residues/regions upon AGO2 phosphorylation at Y393. Analysis of individual residue RMSDs between non-phosphorylated and phosphorylated Y393 AGO2. MDS of specific residues, R110, K112, and E114 located within a β strand on the surface of the N-terminal domain of AGO2 and Y393 located within the L2 linker region.

A

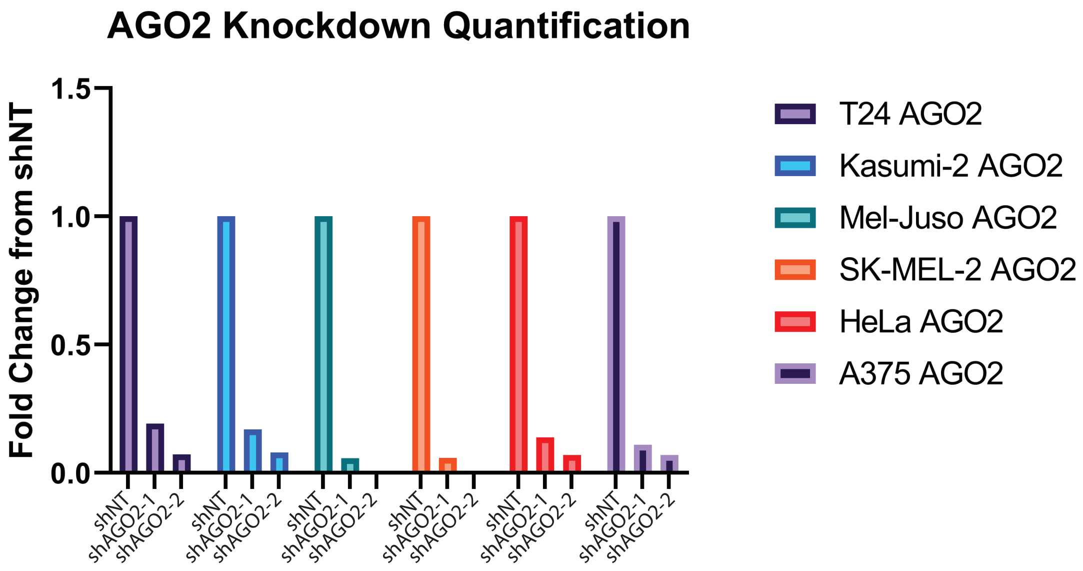

Figure S3. Western blot protein quantification of shAGO2 knockdown presented in Figure 4. Quantification and normalized fold expression change of AGO2 from western blots in Figure 4 following AGO2 knockdown in T24, Kasumi-2, Mel-Juso, SK-MEL-2, HeLa, and A375 cell lines.

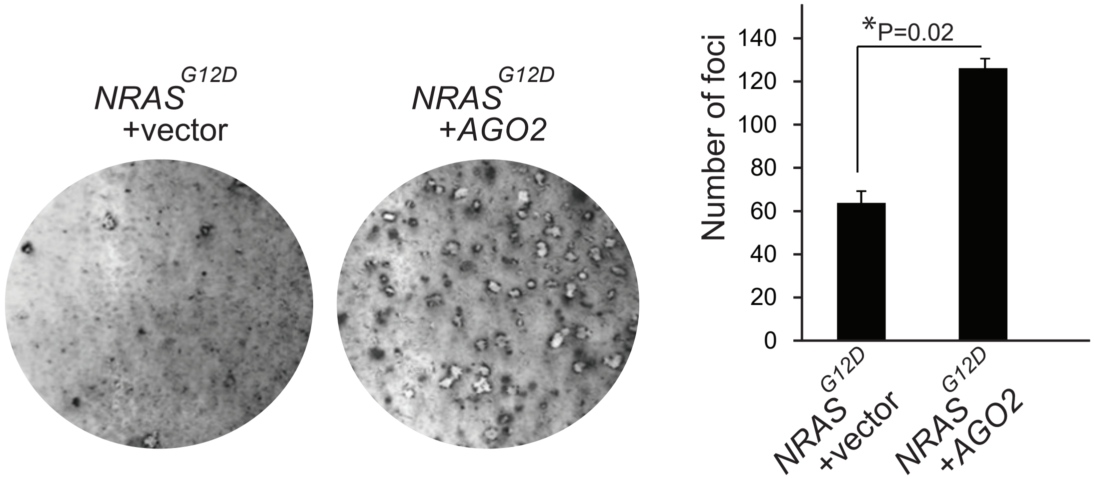

Figure S4. *AGO2* potentiates *RAS*-mediated oncogenesis in NIH-3T3 model. Representative images of foci formation assays using NIH-3T3 cells co-transfected with *NRAS*^{G12D} +/- *AGO2* (left). Quantitation of foci in each experimental condition is shown on the right.

Figure S5

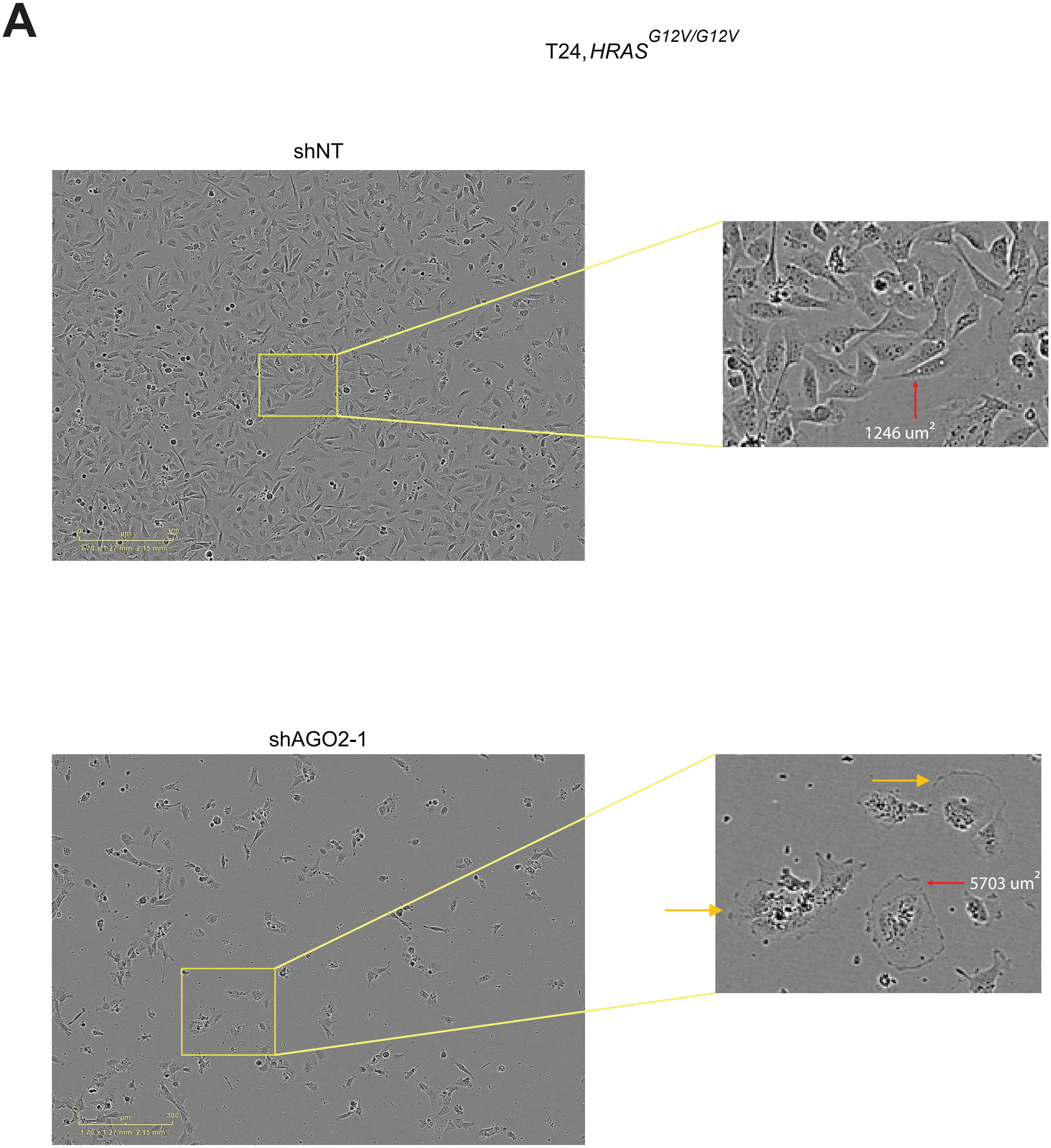

Figure S5. Loss of *AGO2* leads to changes in cell morphology in mutant *HRAS* and *NRAS*-driven cells. Cell morphology changes consistent with cellular senescence in T24 (*HRAS*$^{G12V/G12V}$; **A**) and SK-MEL-2 (*NRAS*$^{Q61R/Q61R}$; **B**) cells following stable knockdown of *AGO2*. Enlarged crops with representative cellular changes with red arrows indicating representative cell sizes, and yellow arrows indicating cells with enlarged/irregular nuclei or cellular flattening following AGO2 knockdown.

Figure S5

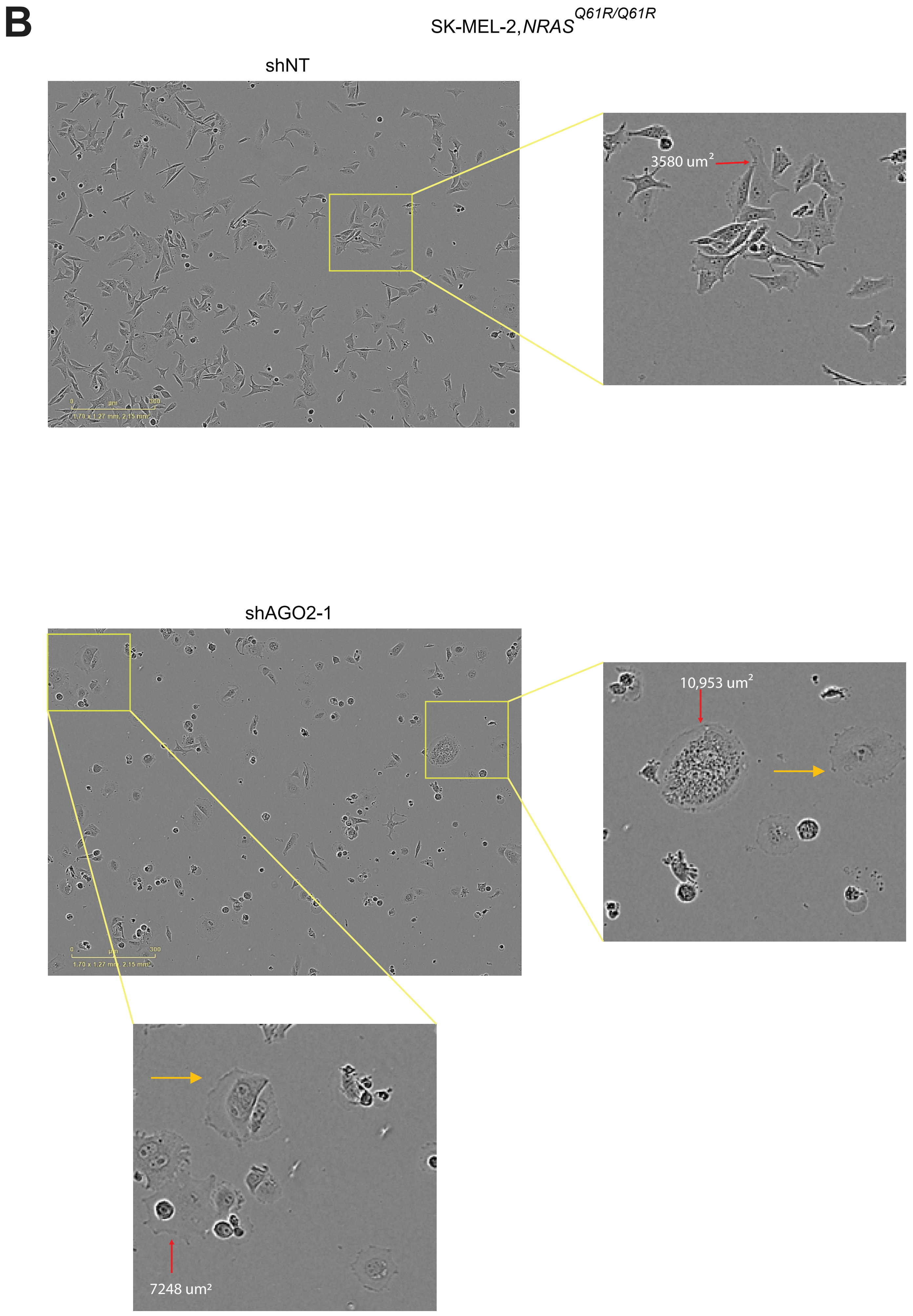
B
SK-MEL-2, NRAS Q61R/Q61R
shNT
3580 um²
shAGO2-1
10,953 um²
7248 um²

A

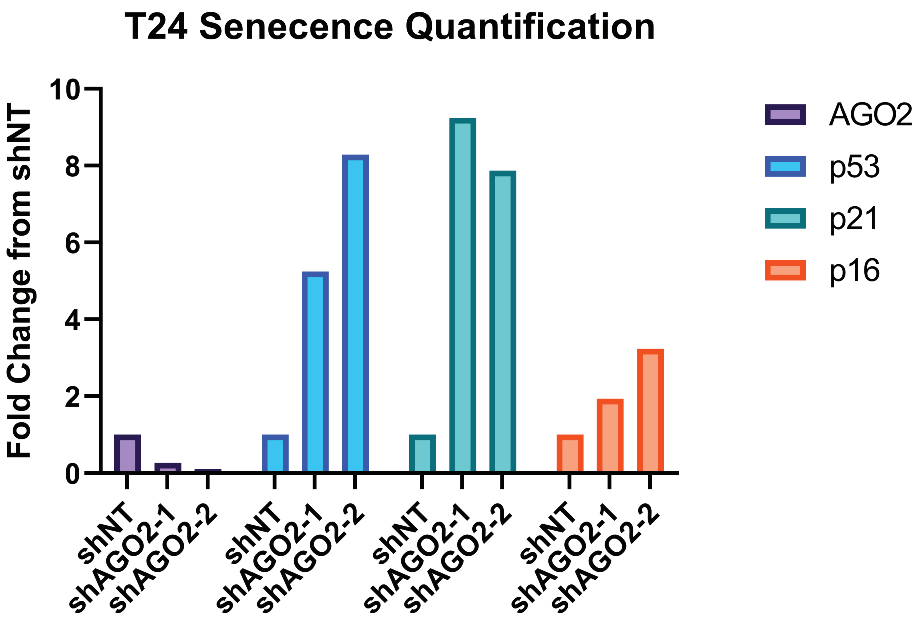

B

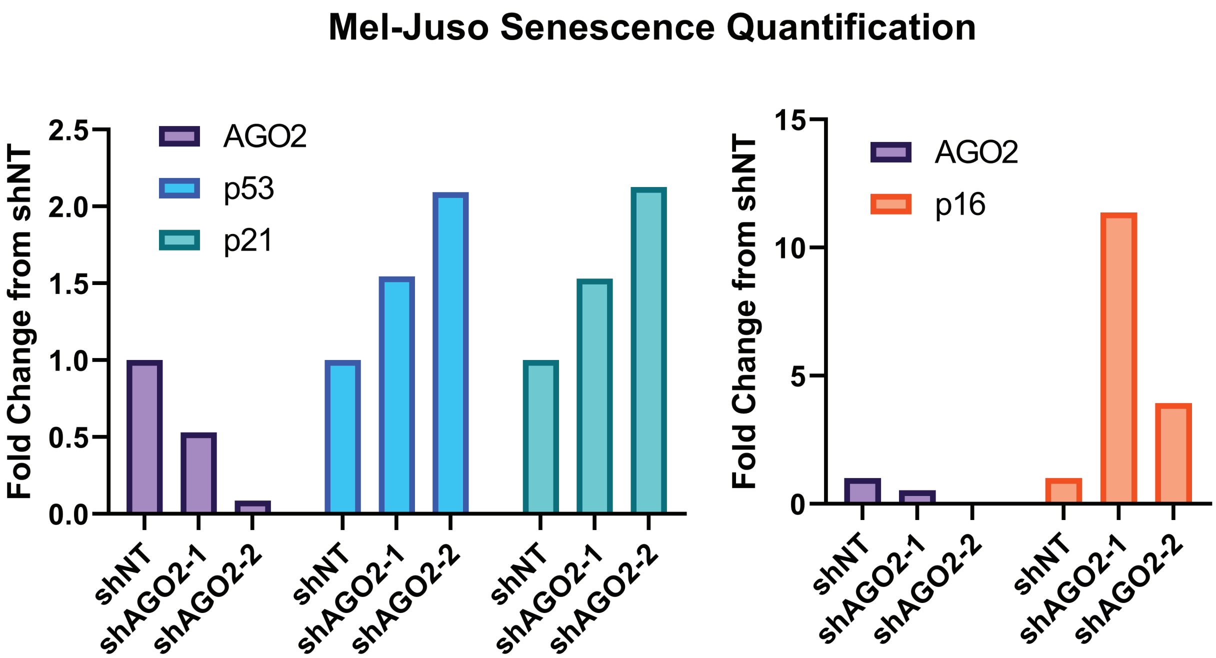

Figure S6. Western blot protein quantification of senescence markers following *AGO2* knockdown in T24 and Mel-Juso cells presented in Figure 5. Quantification and normalized fold expression change of protein from western blots in Figure 5C following AGO2 knockdown in T24 (*HRAS*$^{G12V/G12V}$; **A**) and Mel-Juso (*NRAS*$^{Q61L/WT}$; **B**) cell lines.

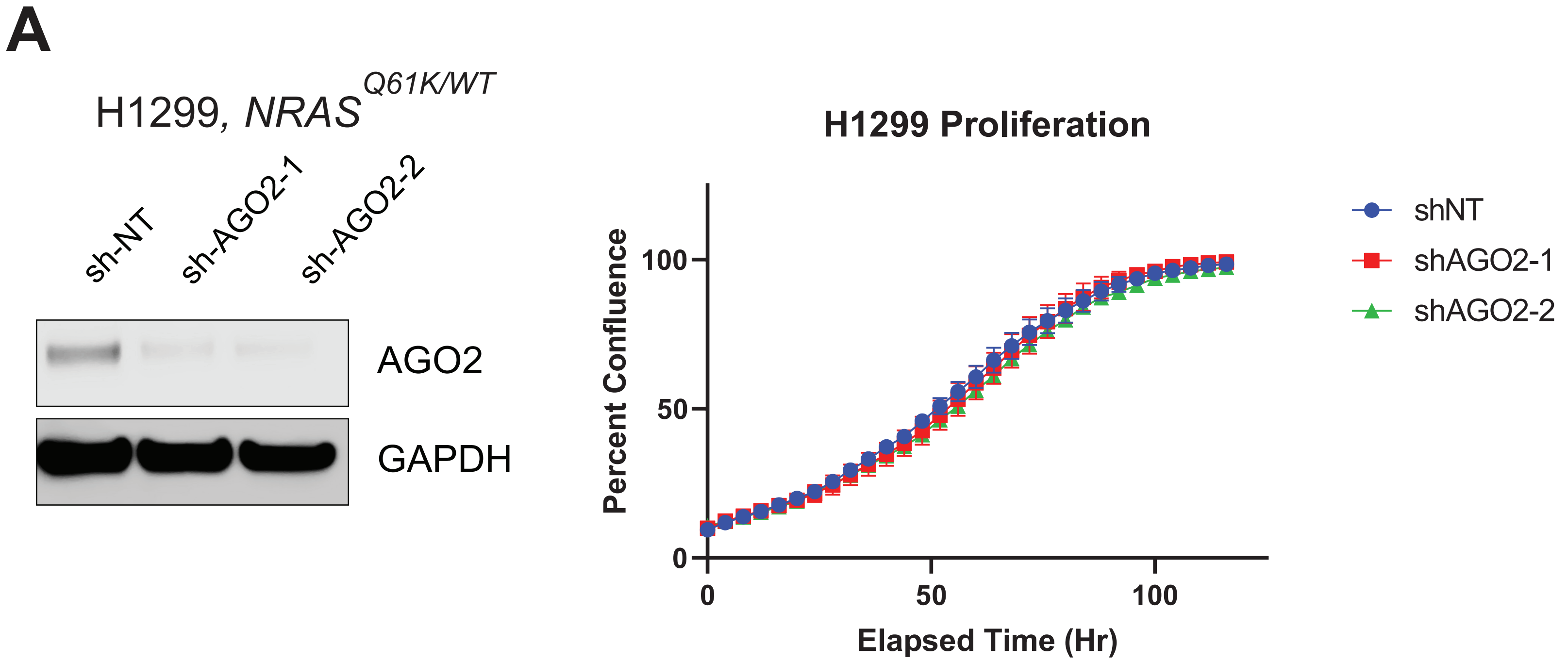

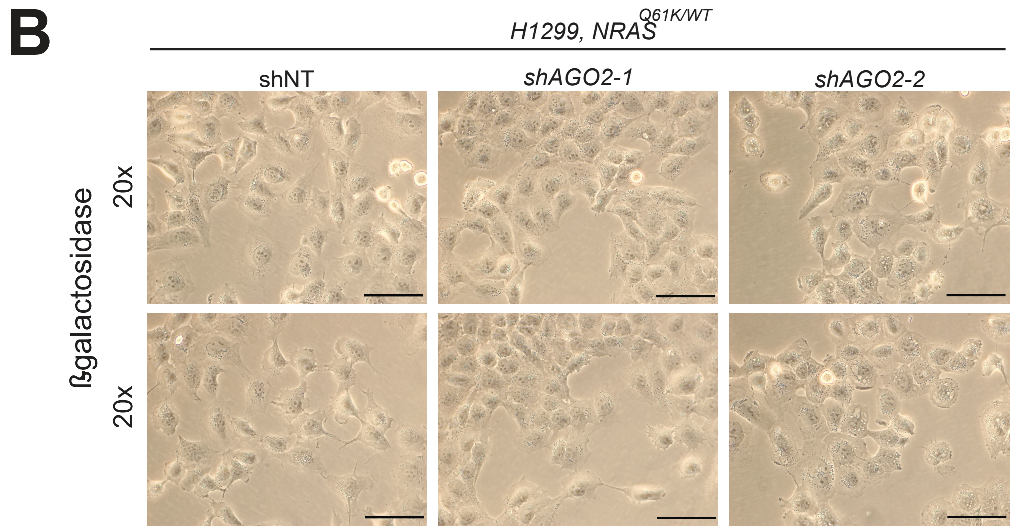

Figure S7. p53 is necessary for oncogene-induced senescence phenotype following loss of *AGO2* in mutant *NRAS* cell lines. (**A**) Immunoblot confirmation of AGO2 knockdown following stable transduction of shRNA transcripts and matched cell proliferation over time in mutant *NRAS*^{Q61k}-driven H1299 cells. (**B**) Representative images following β-galactosidase staining in H1299 cell line following *AGO2* knockdown.

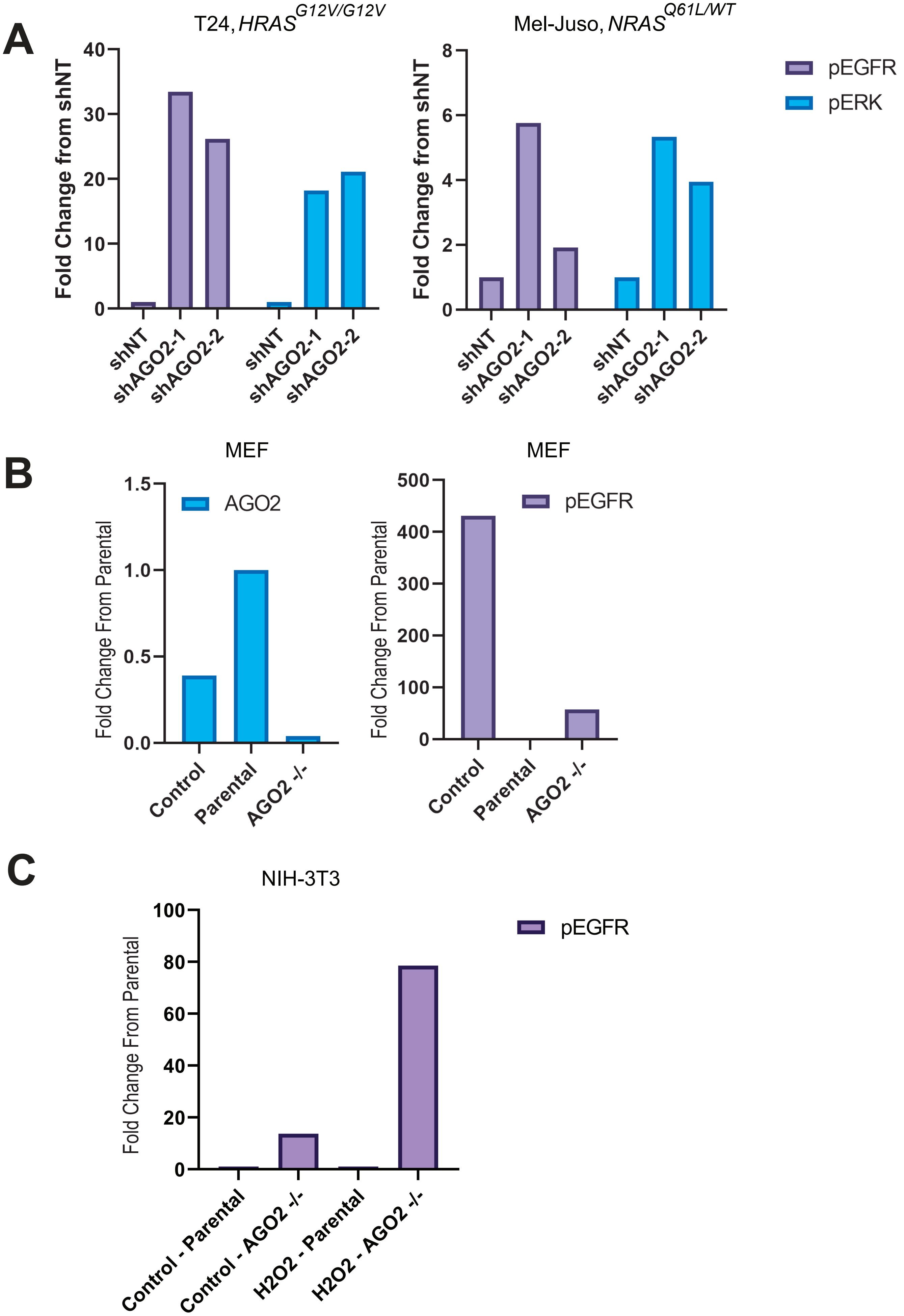
A
T24, HRAS G12V/G12V
Mel-Juso, NRAS Q61L/WT
Fold Change from shNT
40
30
20
10
0
8
6
4
2
shNT
shAGO2-1
shAGO2-2
pEGFR
pERK
B
MEF
1.5
1.0
0.5
0.0
AGO2
Fold Change From Parental
Control
Parental
AGO2 -/-
500
400
300
200
100
pEGFR
C
NIH-3T3
100
80
60
40
20
Control - Parental
Control - AGO2 -/-
H2O2 - Parental
H2O2 - AGO2 -/-

D

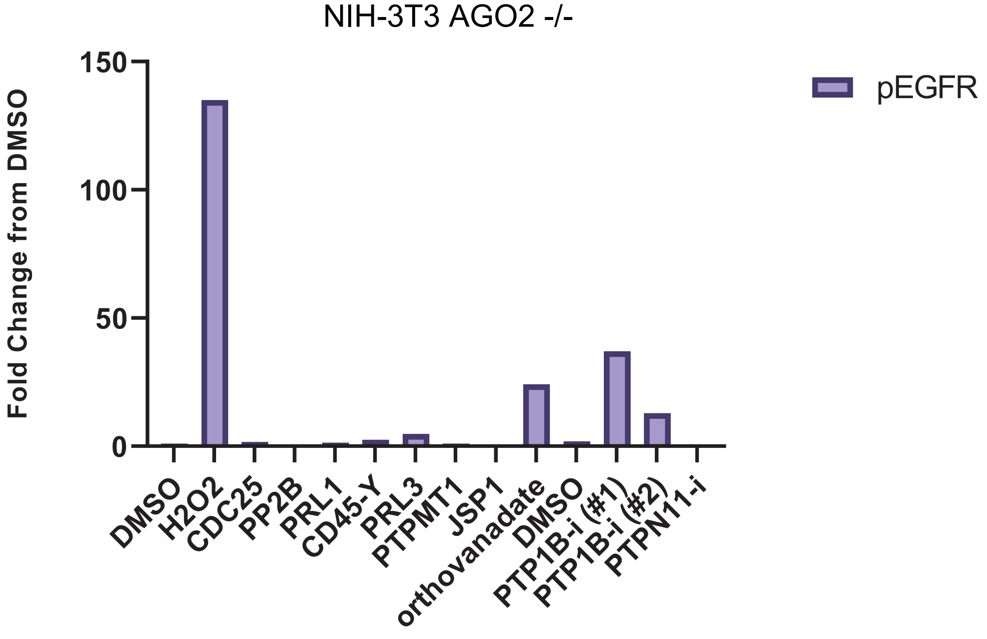

E

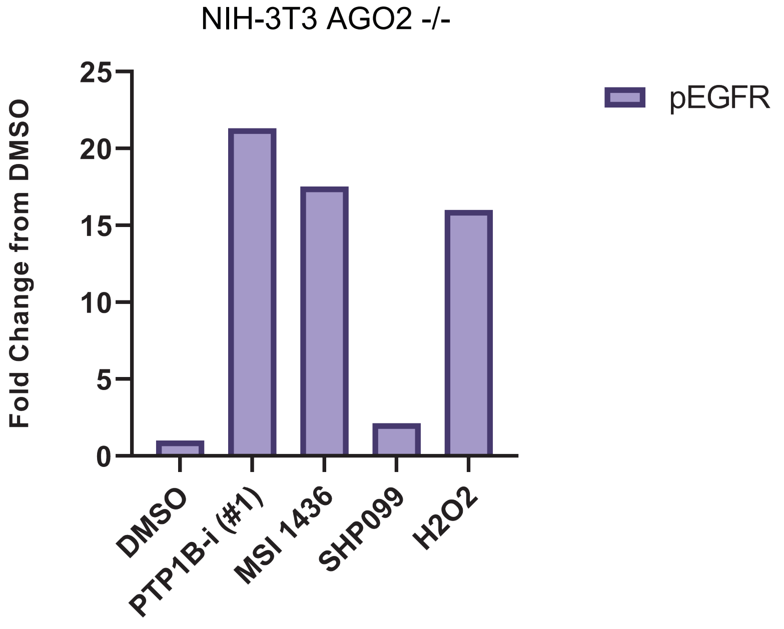

Figure S8. Protein quantification of western blot data presented in Figure 6. (**A**) Quantification and normalized fold expression change of pEGFR and pERK from western blots in Figure 6A following AGO2 knockdown in T24 (*HRAS*$^{G12V/G12V}$) and Mel-Juso (*NRAS*$^{Q61L/WT}$) cell lines. (**B**) quantification and normalized fold expression change of pEGFR and AGO2 in Figure 6B. (**C**) quantification and normalized fold expression change of pEGFR in Figure 6C in NIH-3T3 cells. (**D**) quantification and normalized fold expression change of pEGFR in Figure 6D in NIH-3T3 cells. (**E**) quantification and normalized fold expression change of pEGFR in Figure 6E in NIH-3T3 cells.

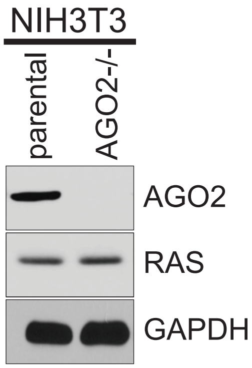

Figure S9. *Ago2* knockout confirmation in NIH-3T3 cells.

Immunoblot confirmation of AGO2 knockout.

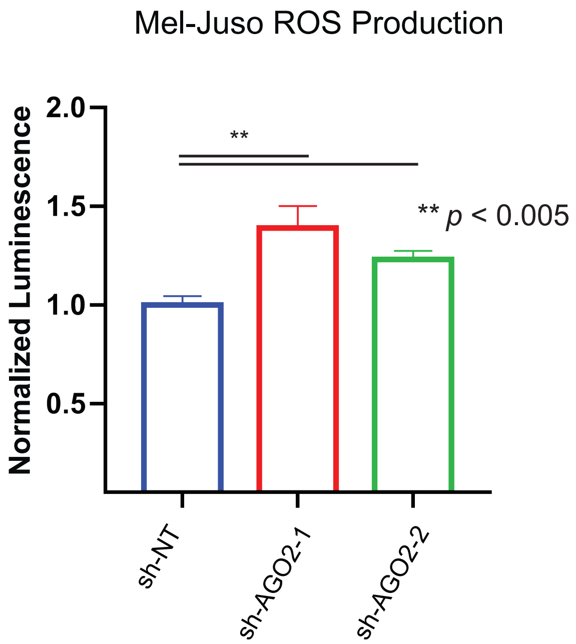

Figure S10. *AGO2* knockdown induces production of reactive oxygen species in mutant *NRAS*-driven cell lines. Normalized ROS production in Mel-Juso (*NRAS*$^{Q61L/WT}$) sh*AGO2* knockdown cell lines. Error bars show standard error of mean of 3 replicates and P value was calculated using two sided t-test.
