# Supplementary material for: Argonaute 2 modulates EGFR–RAS signaling to promote mutant HRAS and NRAS-driven malignancies
Source: PNAS Nexus. 2022 Jul 28;1(3):pgac084. doi: 10.1093/pnasnexus/pgac084 (PMC9338400; doi:10.1093/pnasnexus/pgac084)
Supplement: pgac084_Supplemental_Files [file pgac084_supplemental_files.zip › PNASNEXUS-PNASNEXUS-2022-00105-s02.pdf]

Raw Data From Figure 1B: LNCaP HRAS Immunoprecipitation

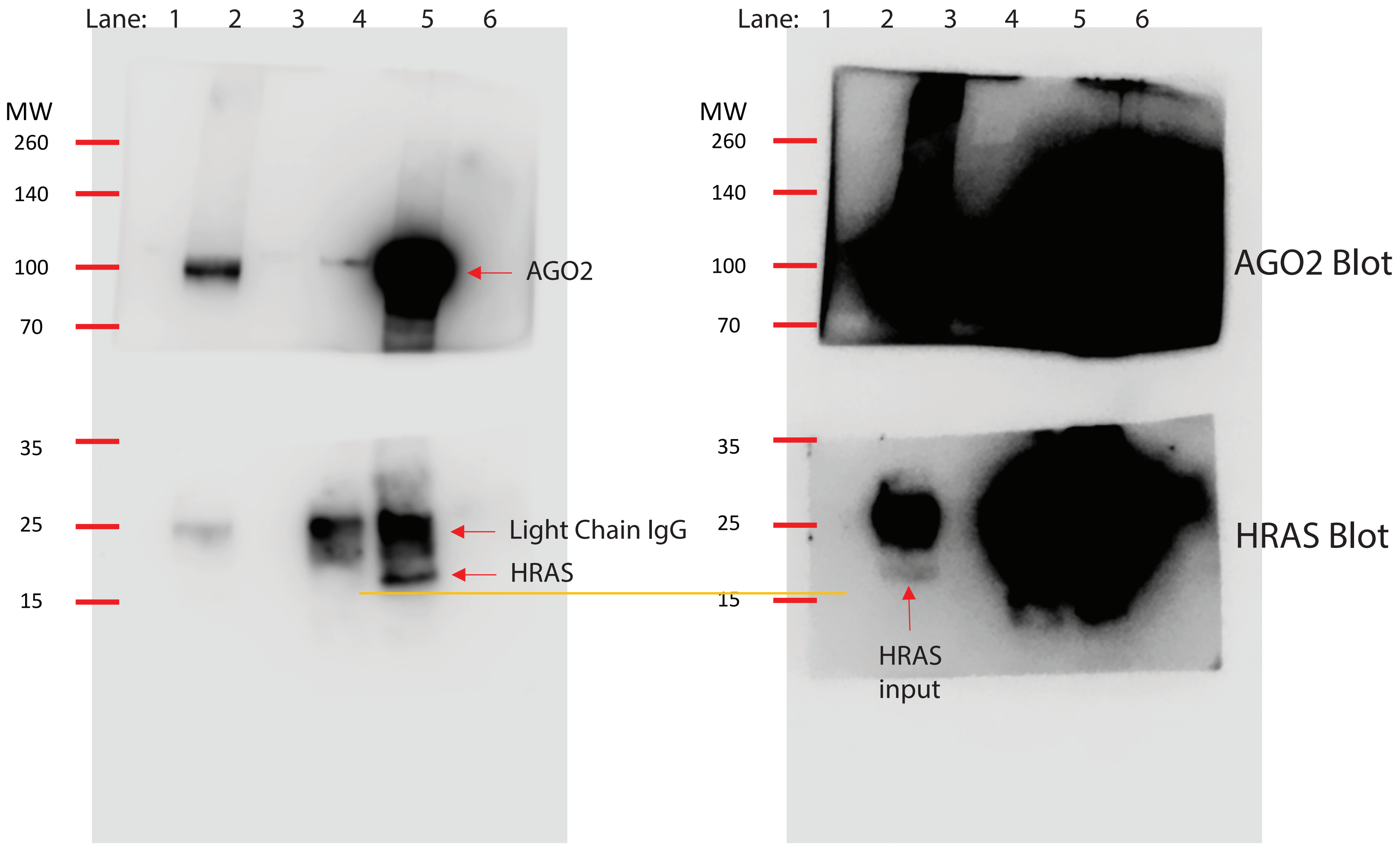

Lane Key:
1. Ladder
2. 5% Input Control
3. Ladder
4. IgG Control
5. HRAS-IP
6. Ladder

Figure R1. Raw western blot images from Main Figure 1b and 1c. Uncropped raw western blot data at various exposures included in Main Figure 1.

Raw Data From Figure 1B: HeLa HRAS Immunoprecipitation

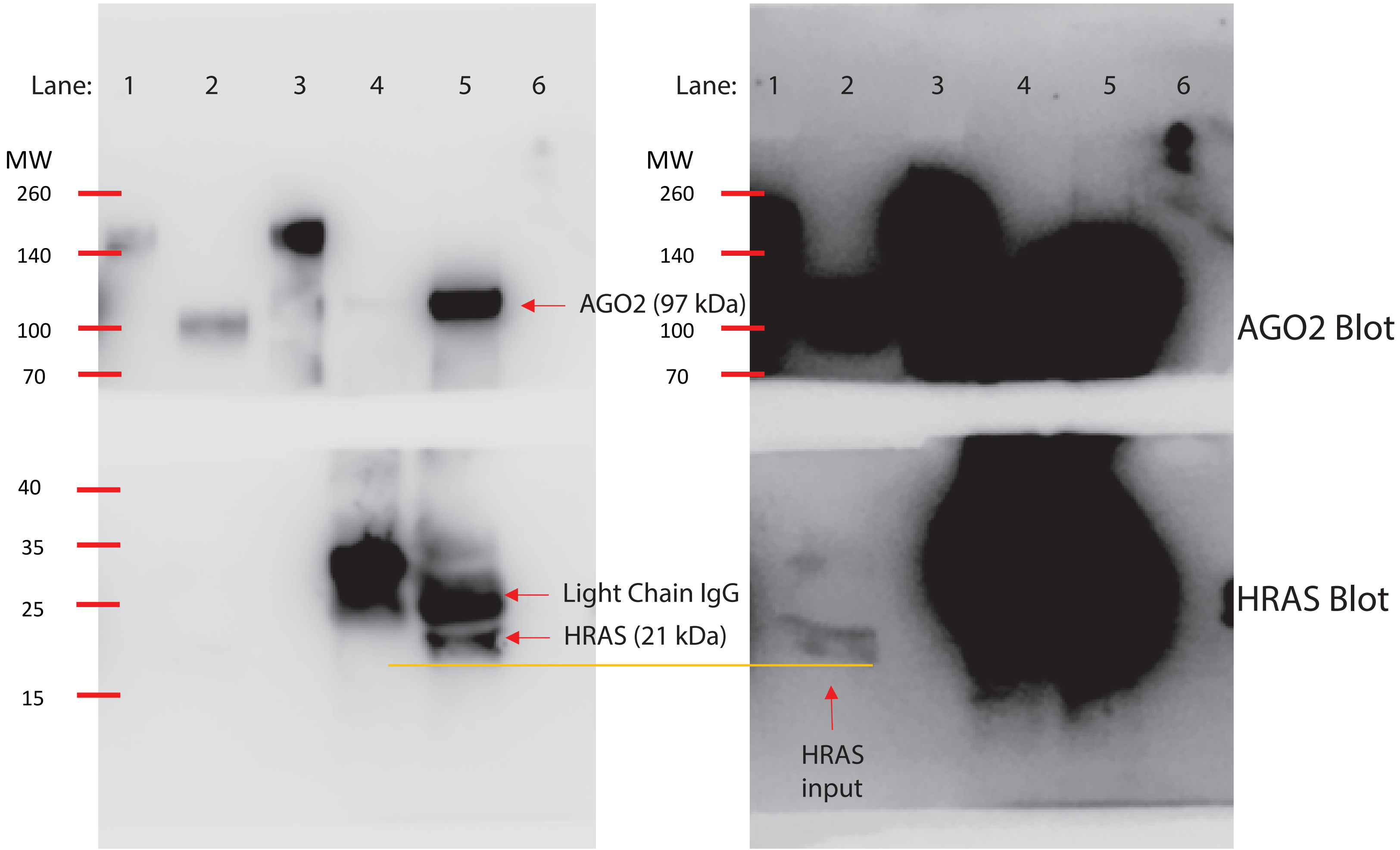

Lane Key:
1. Ladder
2. 5% Input Control
3. Ladder
4. IgG Control
5. HRAS-IP
6. Ladder

Figure R1. Raw western blot images from Main Figure 1b and 1c. Uncropped raw western blot data at various exposures included in Main Figure 1.

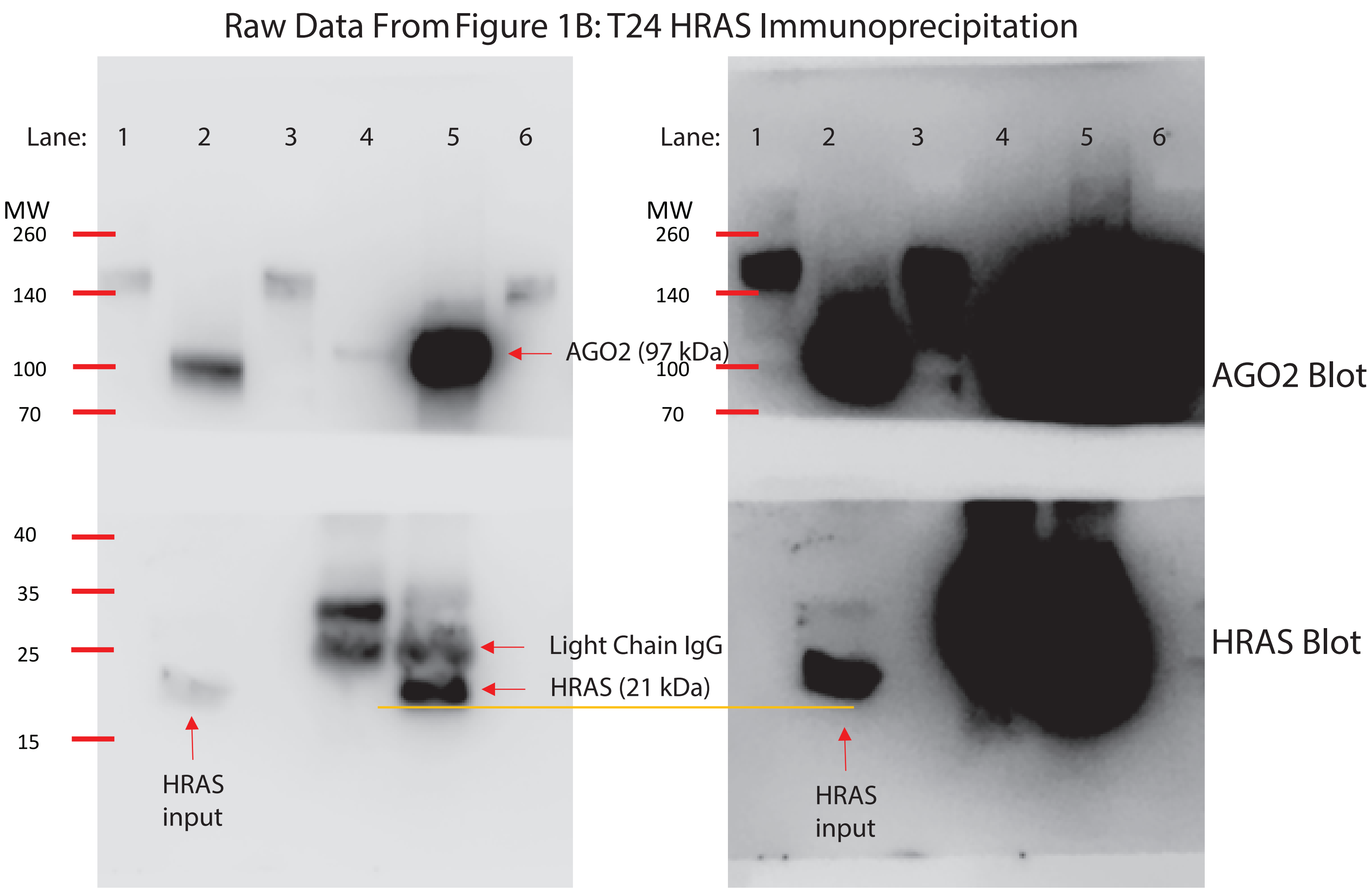

Lane Key:
1. Ladder
2. 5% Input Control
3. Ladder
4. IgG Control
5. HRAS-IP
6. Ladder

Figure R1. Raw western blot images from Main Figure 1b and 1c. Uncropped raw western blot data at various exposures included in Main Figure 1.

Raw Data From Figure 1C: MCF7 NRAS Immunoprecipitation

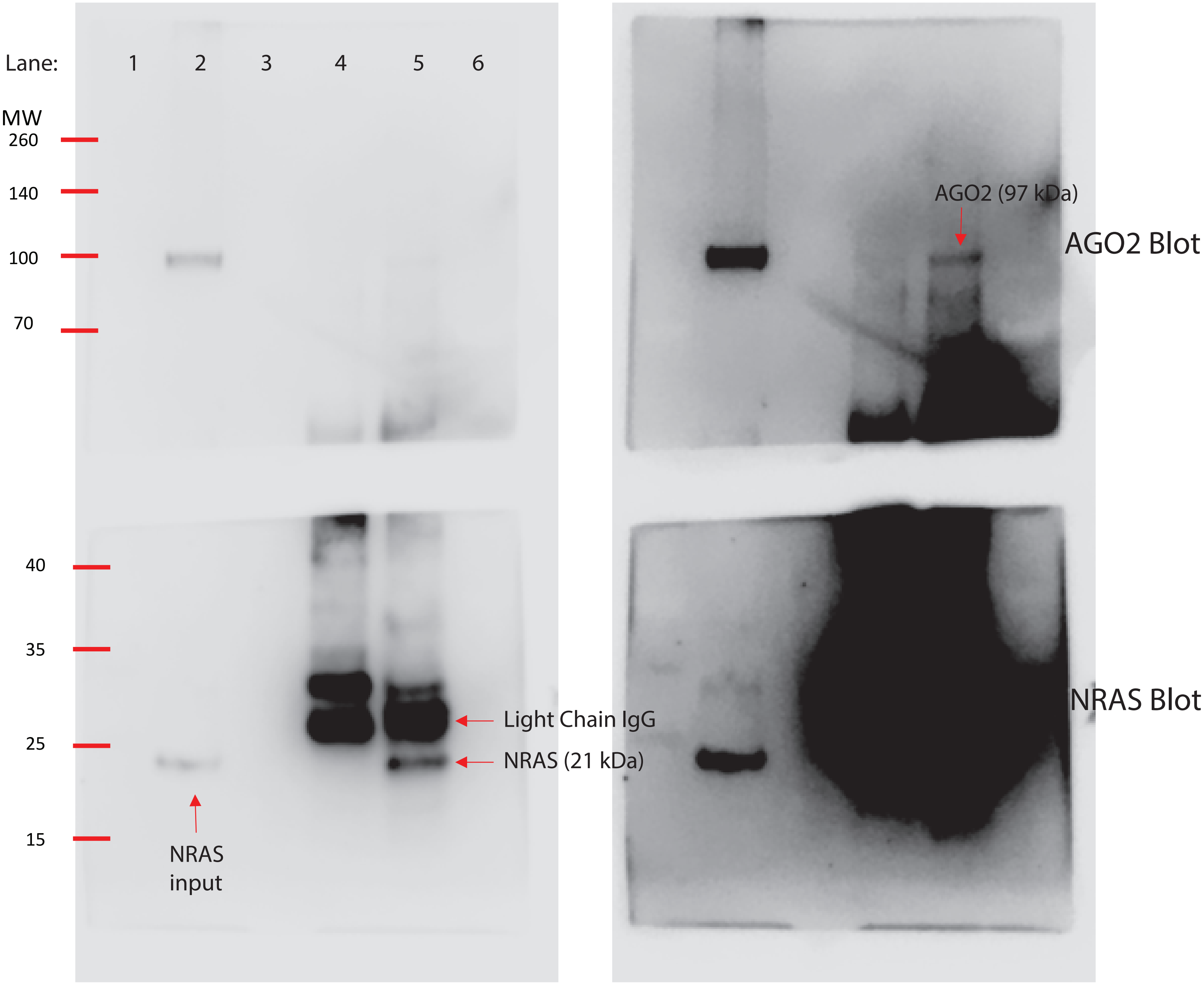

Lane Key:
1. Ladder
2. 5% Input Control
3. Ladder
4. IgG Control
5. NRAS-IP
6. Ladder

Figure R1. Raw western blot images from Main Figure 1b and 1c. Uncropped raw western blot data at various exposures included in Main Figure 1.

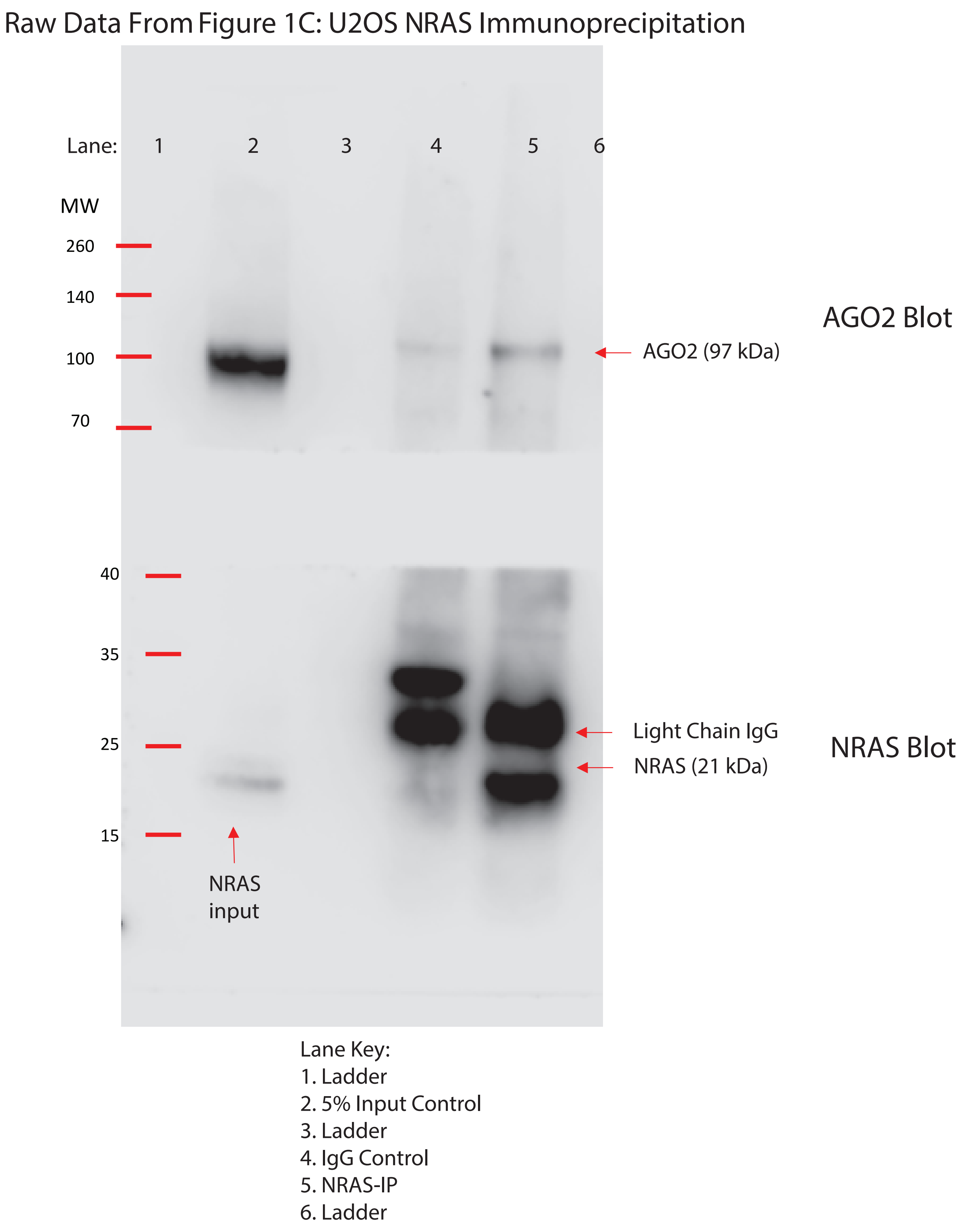

Figure R1. Raw western blot images from Main Figure 1b and 1c. Uncropped raw western blot data at various exposures included in Main Figure 1.

Raw Data From Figure 1C: H1299 NRAS Immunoprecipitation

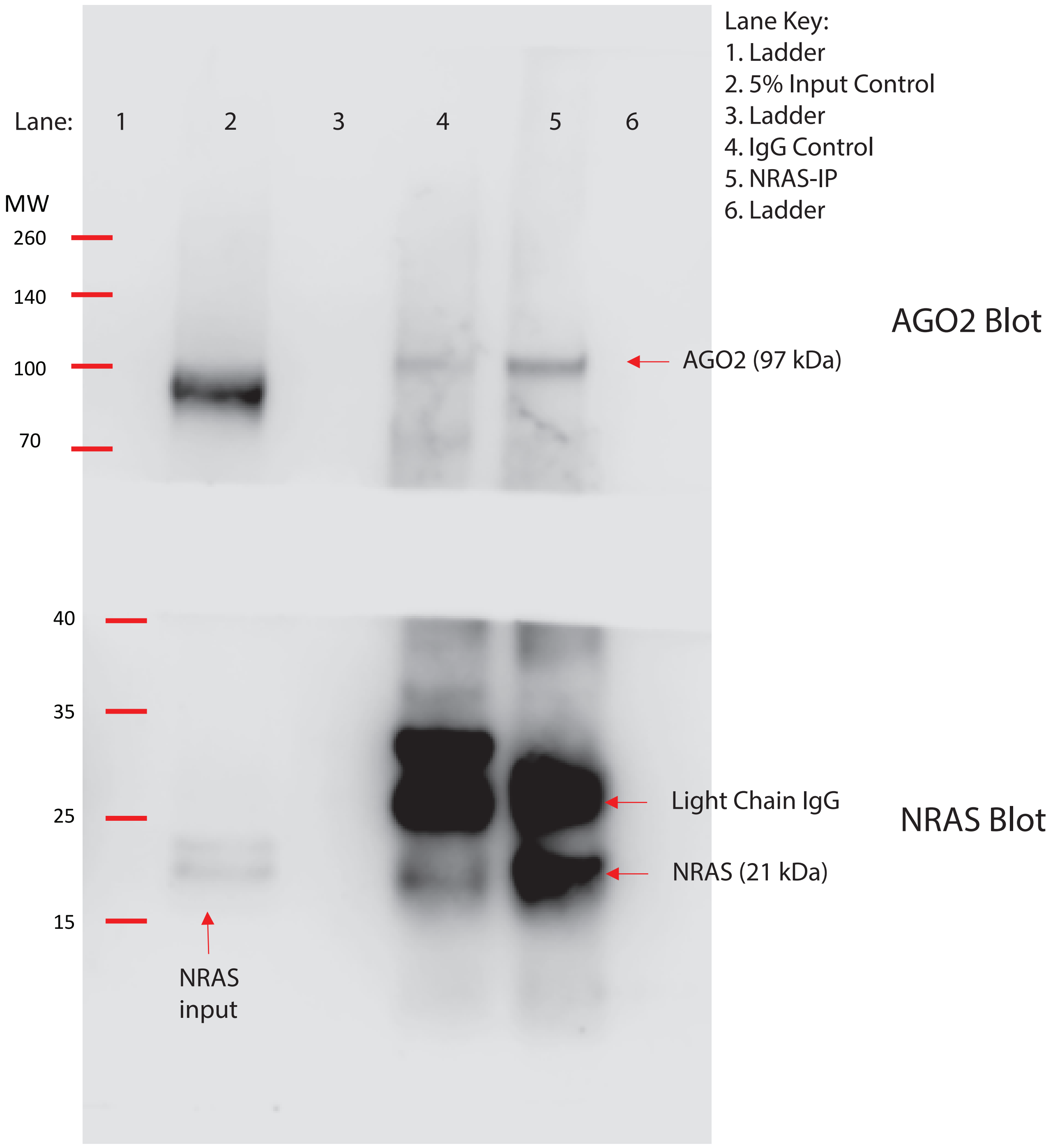

Figure R1. Raw western blot images from Main Figure 1b and 1c. Uncropped raw western blot data at various exposures included in Main Figure 1.

Raw Data From Figure 2A: HeLa AGO2 Immunoprecipitation

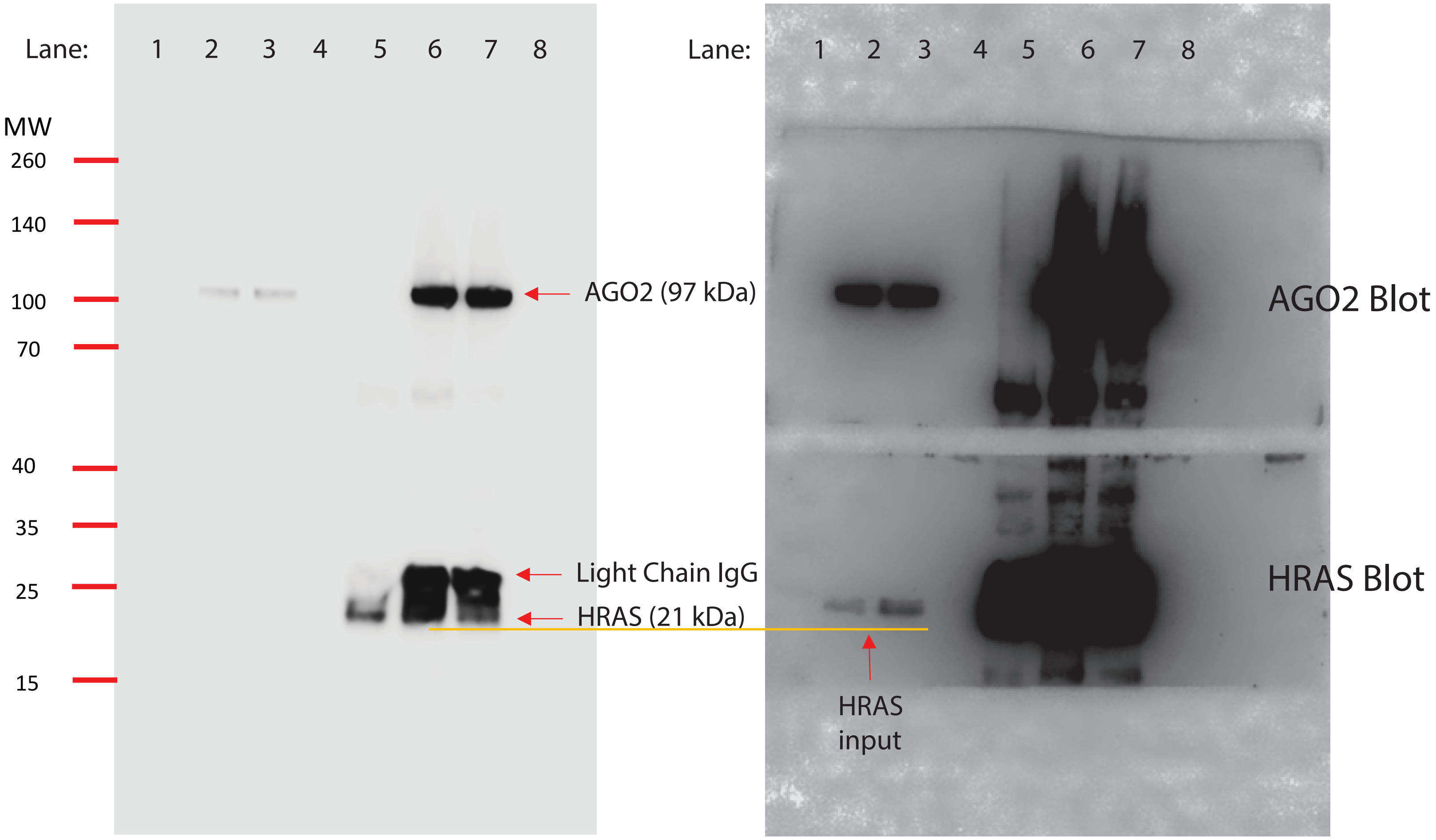

Lane Key:
1. Ladder
2. Serum Starve (5% Input Control)
3. Serum Starve + 5min EGF (5% Input Control)
4. Ladder
5. IgG Control
6. AGO2-IP (Serum Starve)
7. AGO2-IP (Serum Starve + 5min EGF)
8. Ladder

Figure R2 . Raw western blot images from Main Figure 2. Uncropped raw western blot data at various exposures included in Main Figure 2.

Raw Data From Figure 2B: T24 AGO2 Immunoprecipitation

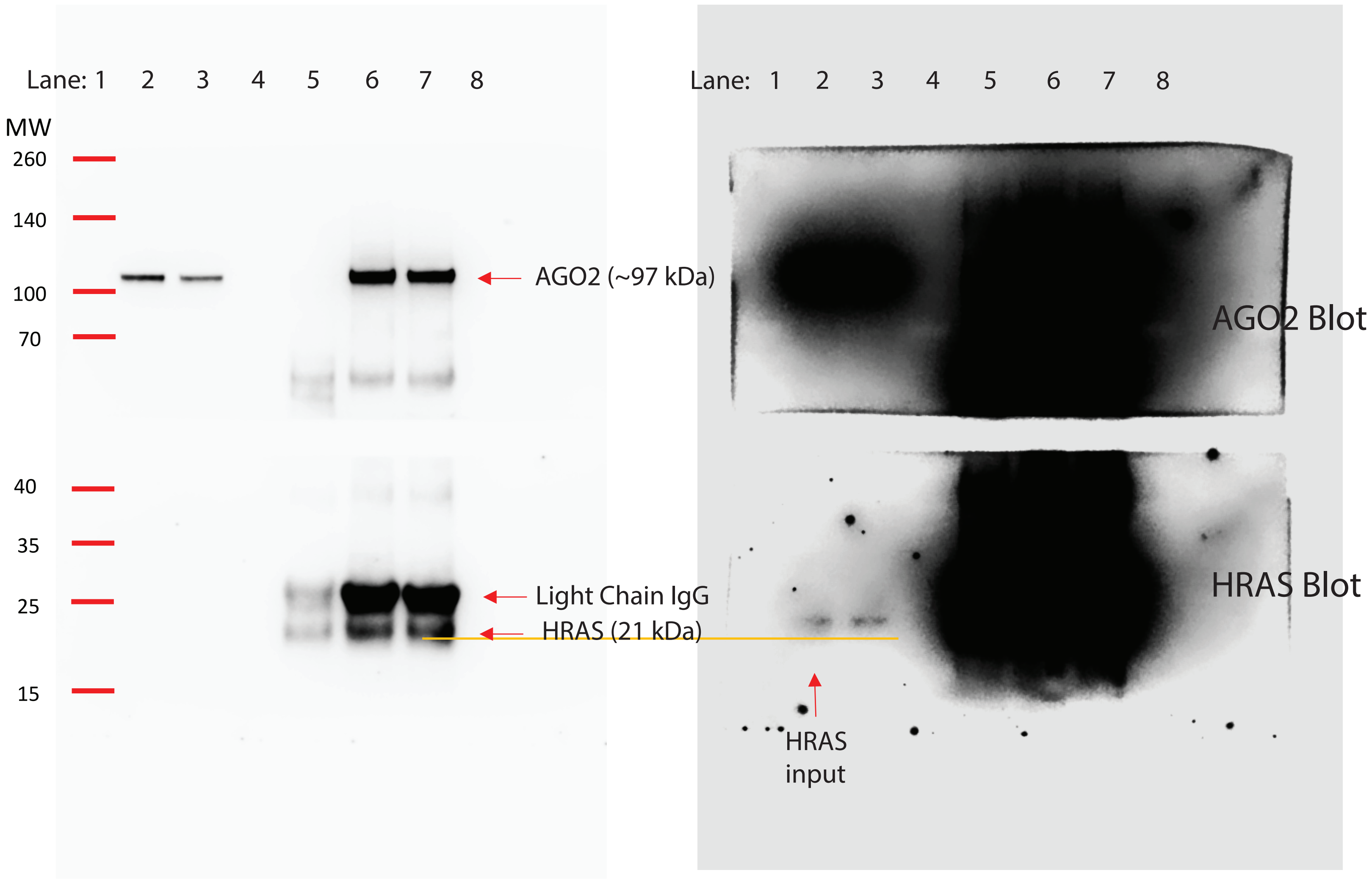

Lane Key:
1. Ladder
2. Serum Starve (5% Input Control)
3. Serum Starve + 5min EGF (5% Input Control)
4. Ladder
5. IgG Control
6. AGO2-IP (Serum Starve)
7. AGO2-IP (Serum Starve + 5min EGF)
8. Ladder

Figure R2 . Raw western blot images from Main Figure 2. Uncropped raw western blot data at various exposures included in Main Figure 2.

Raw Data From Figure 2C: MCF7 AGO2 Immunoprecipitation

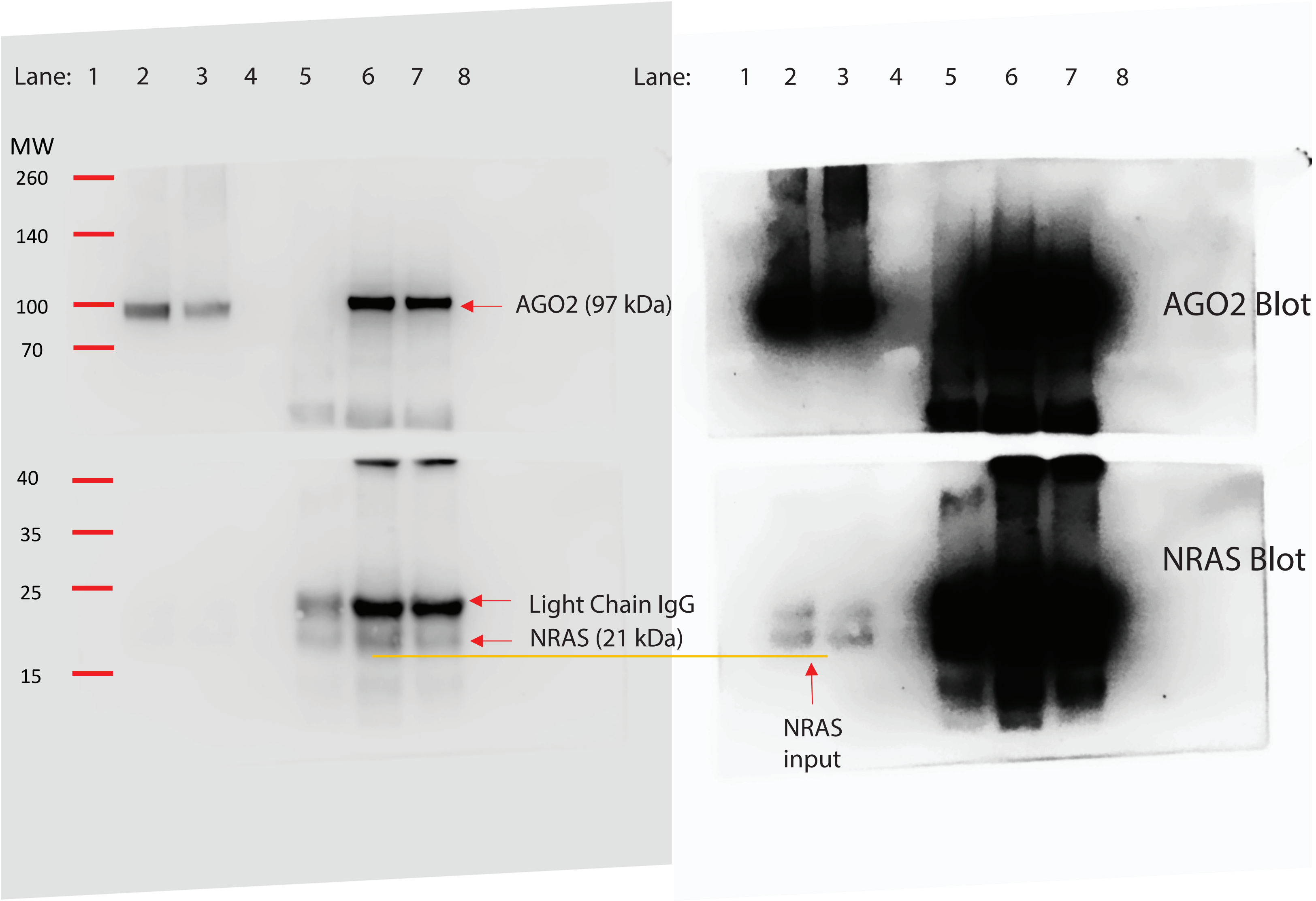

Lane Key:
1. Ladder
2. Serum Starve (5% Input Control)
3. Serum Starve + 5min EGF (5% Input Control)
4. Ladder
5. IgG Control
6. AGO2-IP (Serum Starve)
7. AGO2-IP (Serum Starve + 5min EGF)
8. Ladder

Figure R2 . Raw western blot images from Main Figure 2. Uncropped raw western blot data at various exposures included in Main Figure 2.

Raw Data From Figure 2D: Mel-Juso AGO2 Immunoprecipitation

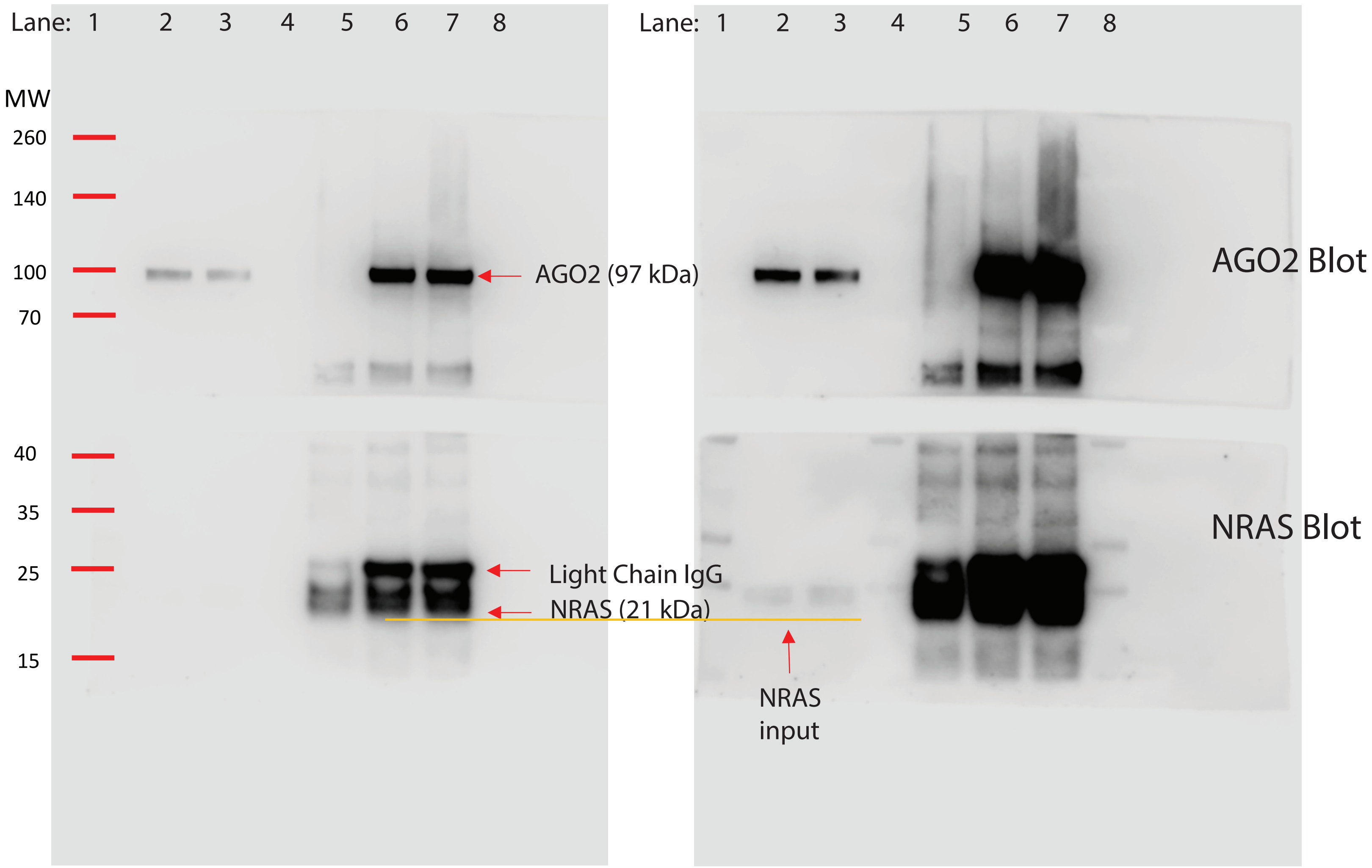

Lane Key:
1. Ladder
2. Serum Starve (5% Input Control)
3. Serum Starve + 5min EGF (5% Input Control)
4. Ladder
5. IgG Control
6. AGO2-IP (Serum Starve)
7. AGO2-IP (Serum Starve + 5min EGF)
8. Ladder

Figure R2 . Raw western blot images from Main Figure 2. Uncropped raw western blot data at various exposures included in Main Figure 2.

Raw Data From Figure 3A: U2OS, FLAG-AGO2; RAS-IP

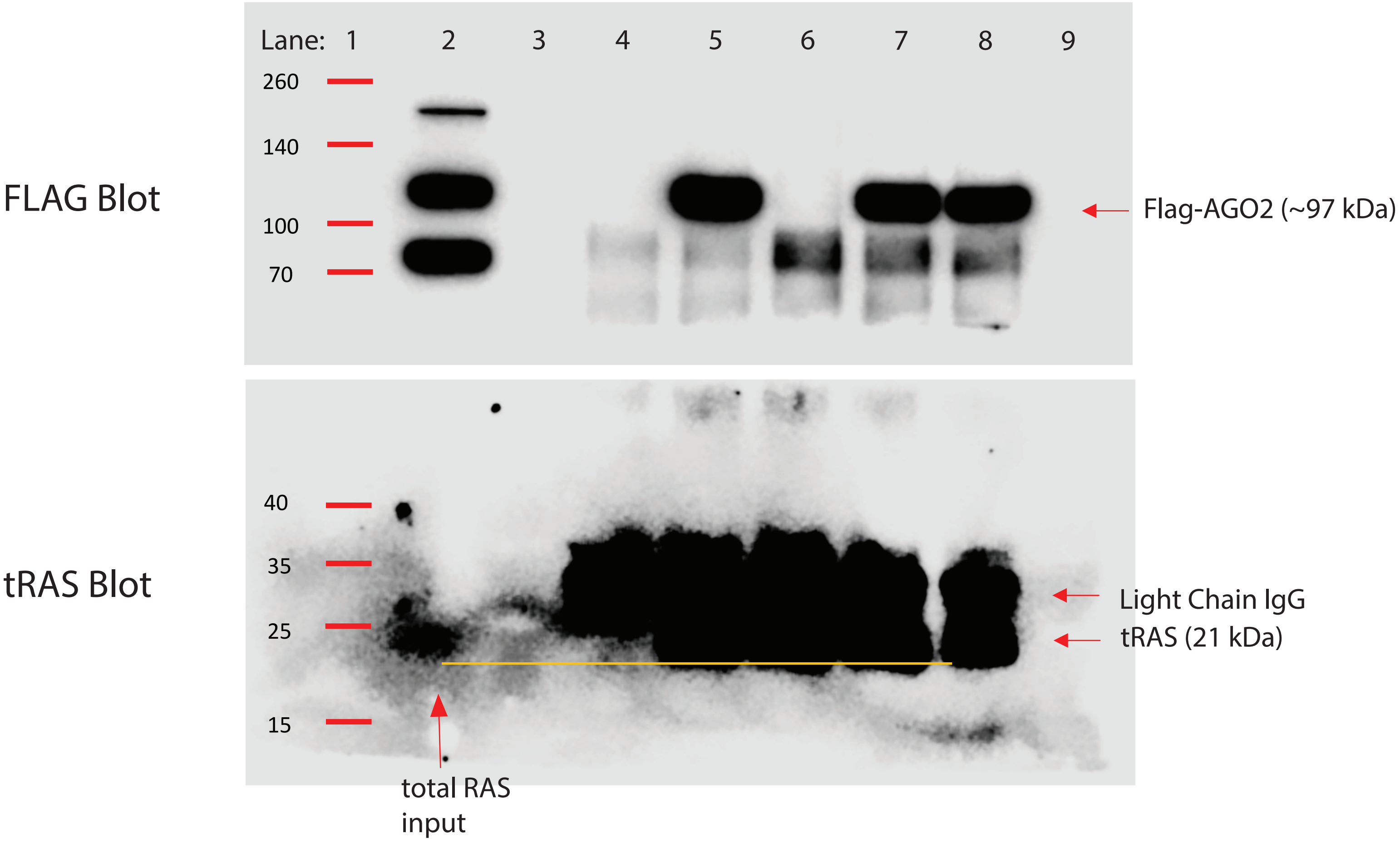

Lane Key:
1. Ladder
2. 5% Input Control
3. Ladder
4. IgG Control
5. RAS-IP (Flag-AGO2 WT Serum Starve)
6. RAS-IP (Flag-AGO2 WT Serum Starve + 5min EGF)
7. RAS-IP (Flag-AGO2 Y393F Serum Starve)
8. RAS-IP (Flag-AGO2 Y393F Serum Starve + 5min EGF)
9. Ladder

Figure R3 Raw western blot images from Main Figure 3. Uncropped raw western blot data at various exposures included in Main Figure 3.

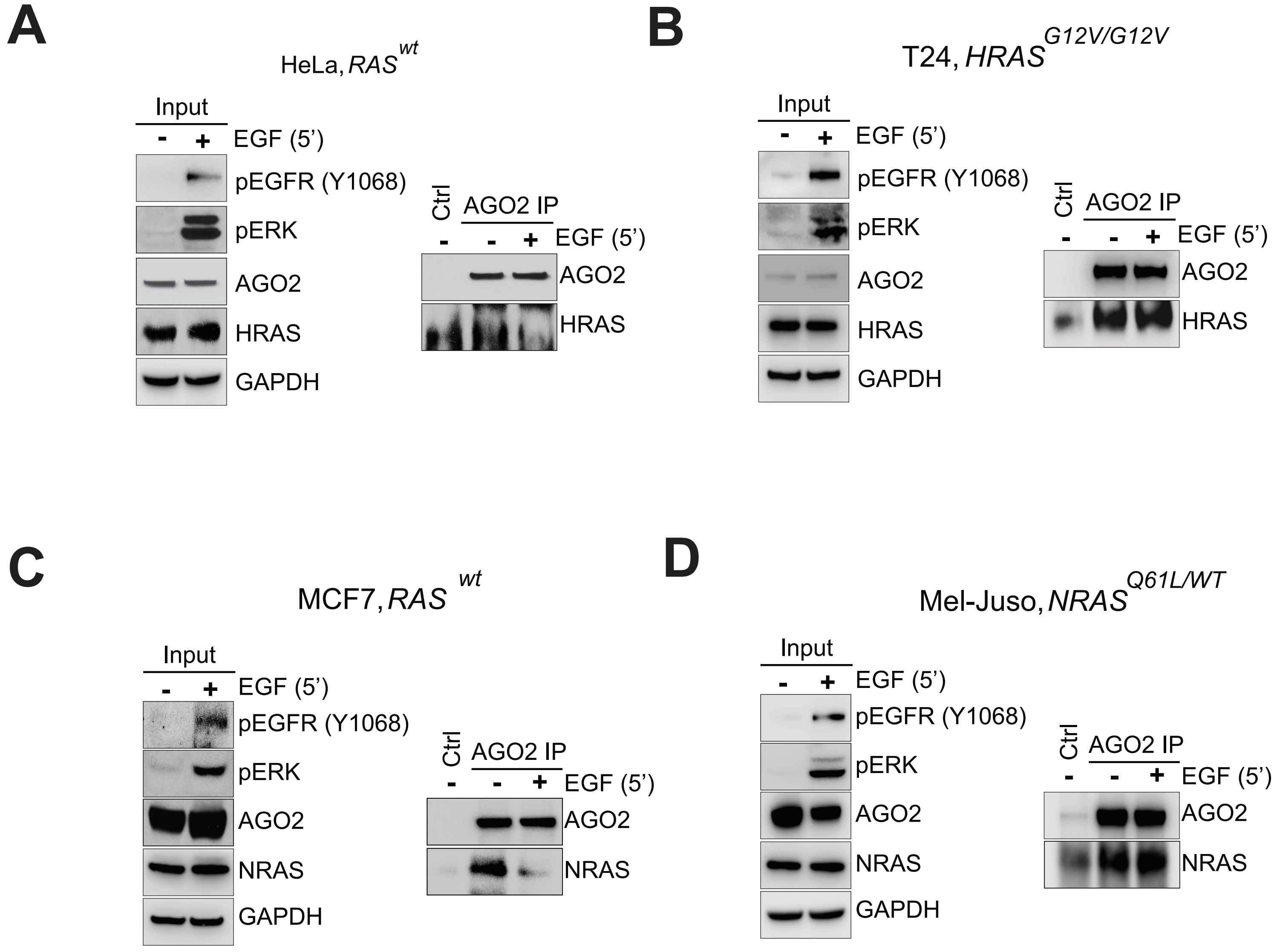

Figure R4 Select Replicates of Main Figure 2. (**A**) IP of endogenous AGO2 upon EGF stimulation (5') in HeLa cell line expressing WT HRAS followed by immunoblot analysis of HRAS-AGO2 interaction. Ctrl lane on IP represents matched isotype control. (**B**) Co-IP of endogenous AGO2 following EGF stimulation (5') in T24 cancer cells harboring *HRAS*$^{G12V/G12V}$ mutations, followed by immunoblot analysis of HRAS. Ctrl lane on IP represents matched isotype control. (**C**) Immunoprecipitation of AGO2 following EGF stimulation (5') in MCF7 cell line expressing WT NRAS with immunoblot analysis of the NRAS-AGO2 interaction. Ctrl lane on IP represents matched isotype control. (**D**) Co-IP of endogenous AGO2 in Mel-Juso cell with *NRAS*$^{Q61L/WT}$ mutation. Ctrl lane on IP represents matched isotype control. For each cell line, MAPK activation and levels of various proteins are shown as input blots.

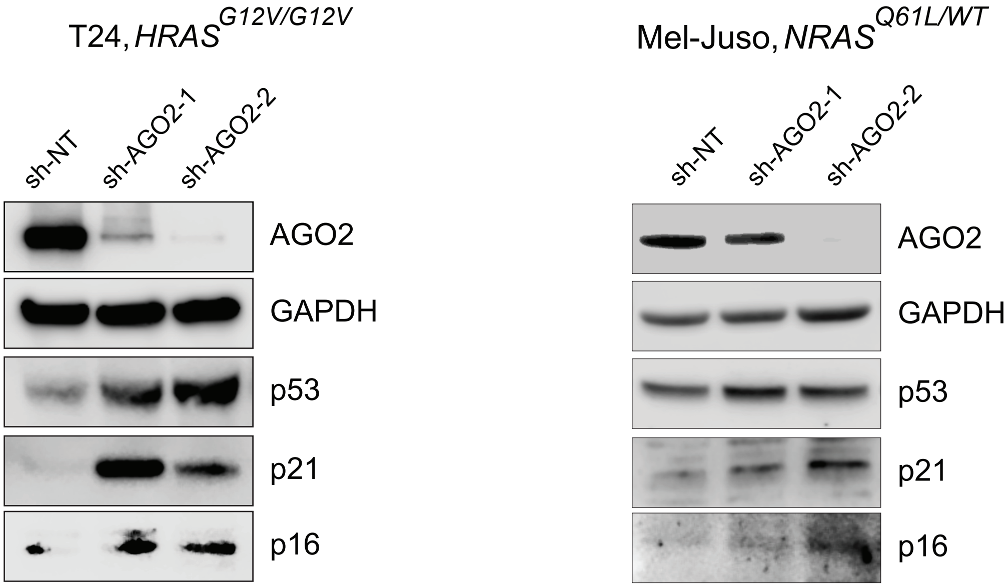

Figure R5 Selected Replicates from Main Figure 5C. Immunoblot blot analysis of senescence markers (p53, p21, and p16) in stable *AGO2* knockdown T24 (*HRAS*$^{G12V/G12V}$) and Mel-Juso (*NRAS*$^{Q61L/WT}$) cells.

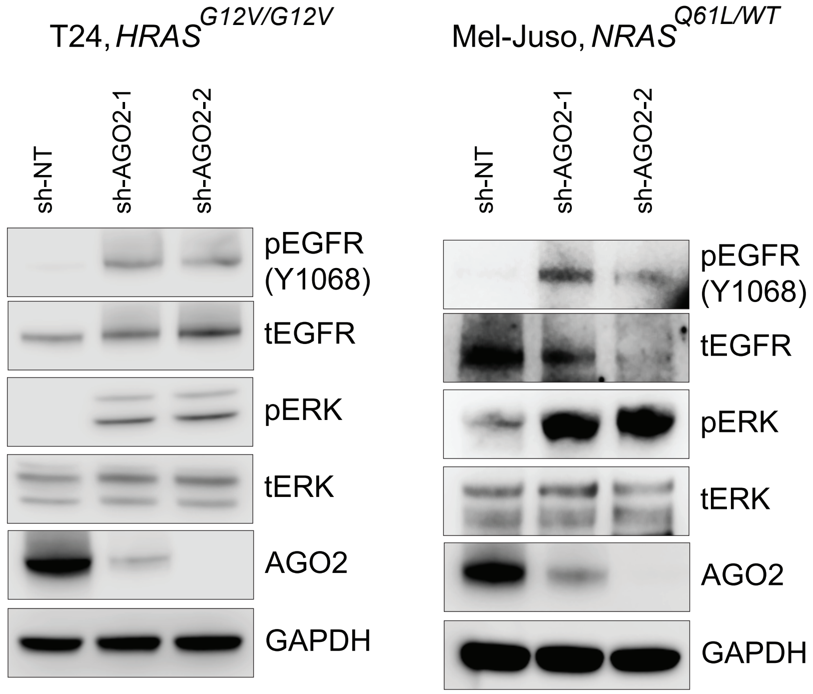

Figure R6 Selected Replicates from Main Figure 6A. Immunoblot blot analysis of pERK and pEGFR (Y1068) induction with stable *AGO2* knockdown in T24 (*HRAS*$^{G12V/G12V}$) and Mel-Juso (*NRAS*$^{Q61L/WT}$) cells.

Raw Data From Supplemental Figure 1A: LNCaP AGO2 Immunoprecipitation

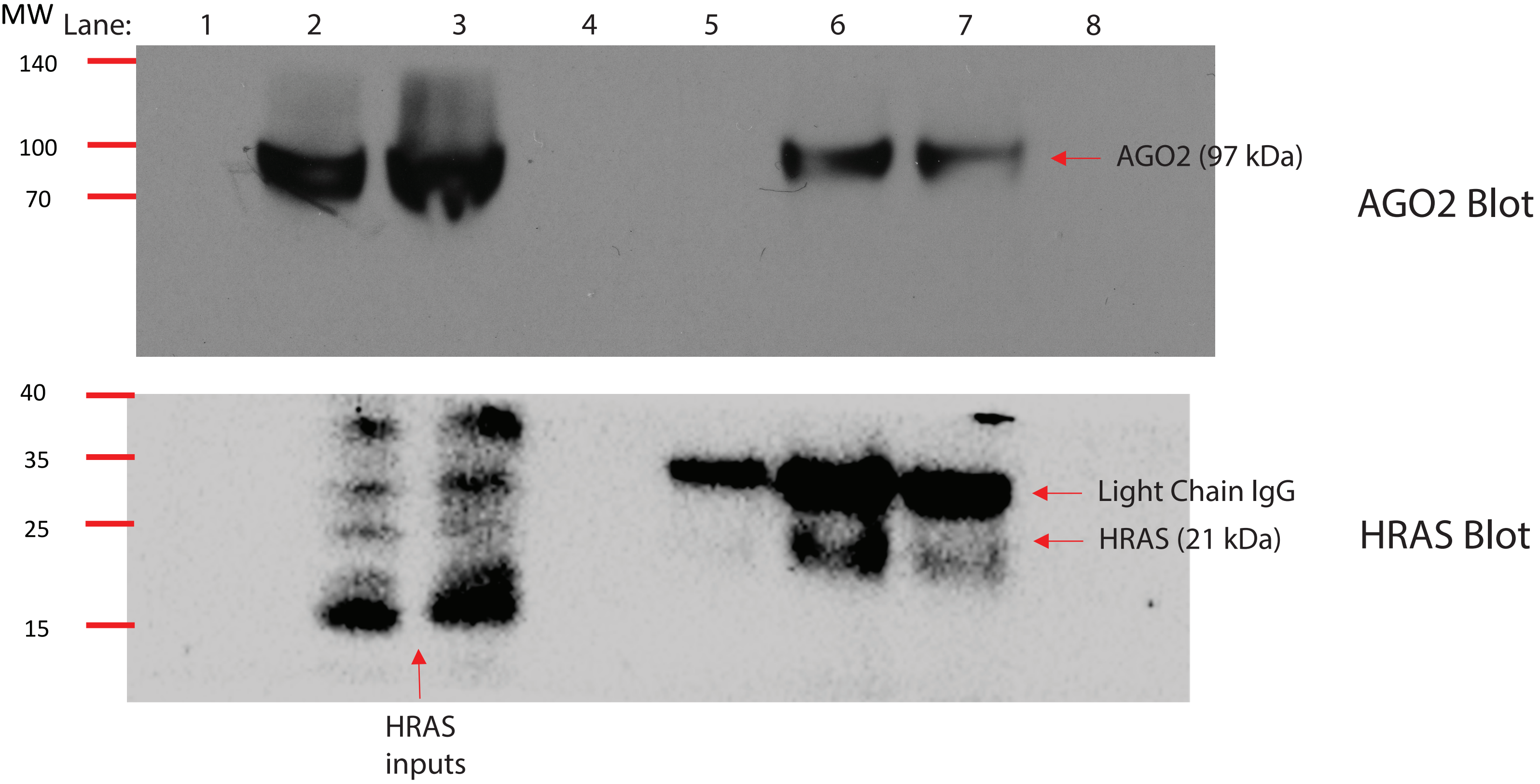

Lane Key:
1. Ladder
2. Serum Starve (5% Input Control)
3. Serum Starve + 5min EGF (5% Input Control)
4. Ladder
5. IgG Control
6. AGO2-IP (Serum Starve)
7. AGO2-IP (Serum Starve + 5min EGF)
8. Ladder

Figure R7 Raw western blot images from Figure S1. Uncropped raw western blot data at various exposures included in Supplemental Figure 1.

Raw Data From Supplemental Figure 1B: Hs578t AGO2 Immunoprecipitation

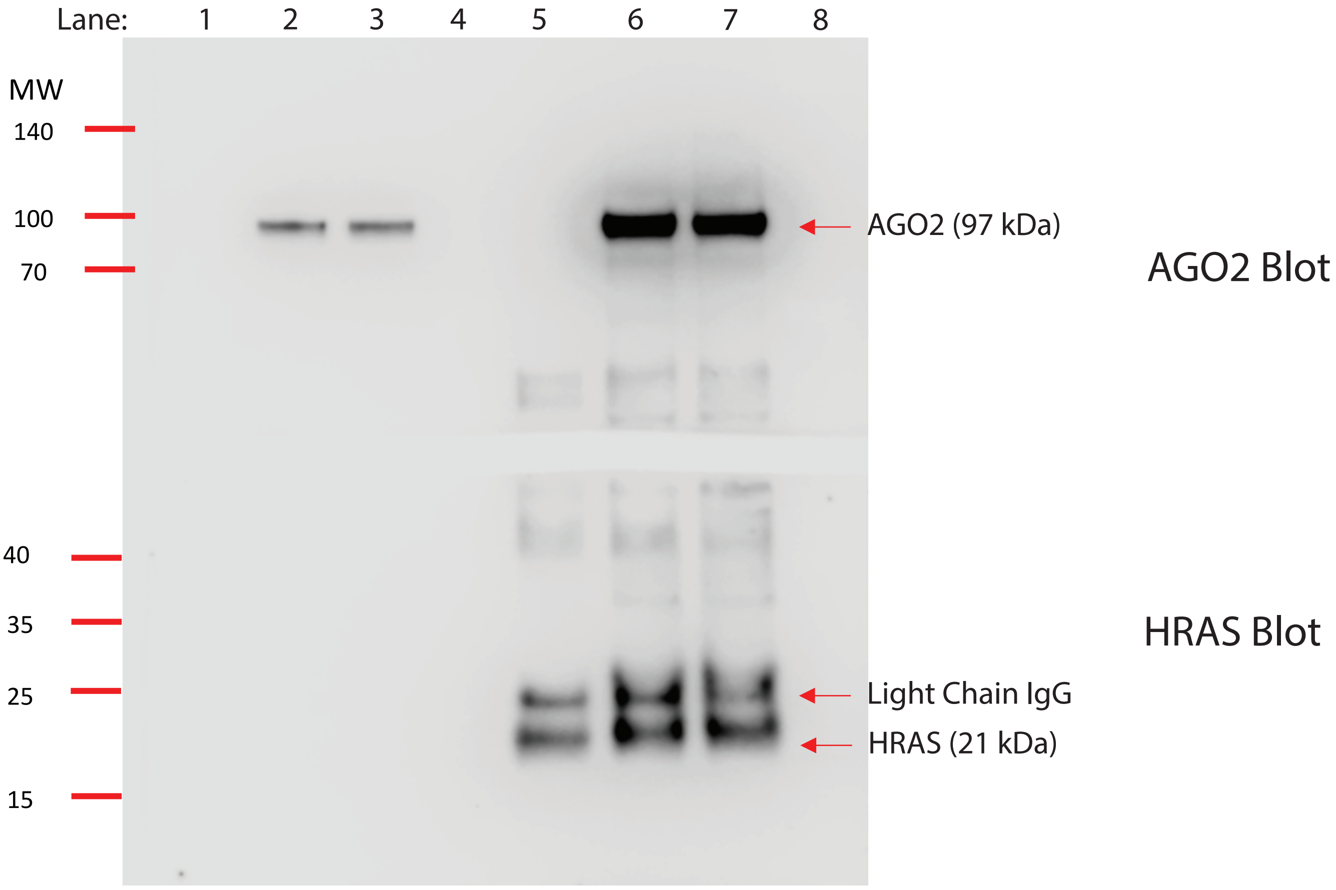

Lane Key:
1. Ladder
2. Serum Starve (5% Input Control)
3. Serum Starve + 5min EGF (5% Input Control)
4. Ladder
5. IgG Control
6. AGO2-IP (Serum Starve)
7. AGO2-IP (Serum Starve + 5min EGF)
8. Ladder

Figure R7 Raw western blot images from Figure S1. Uncropped raw western blot data at various exposures included in Supplemental Figure 1.

Raw Data From Supplemental Figure 1C: HEK293 AGO2 Immunoprecipitation

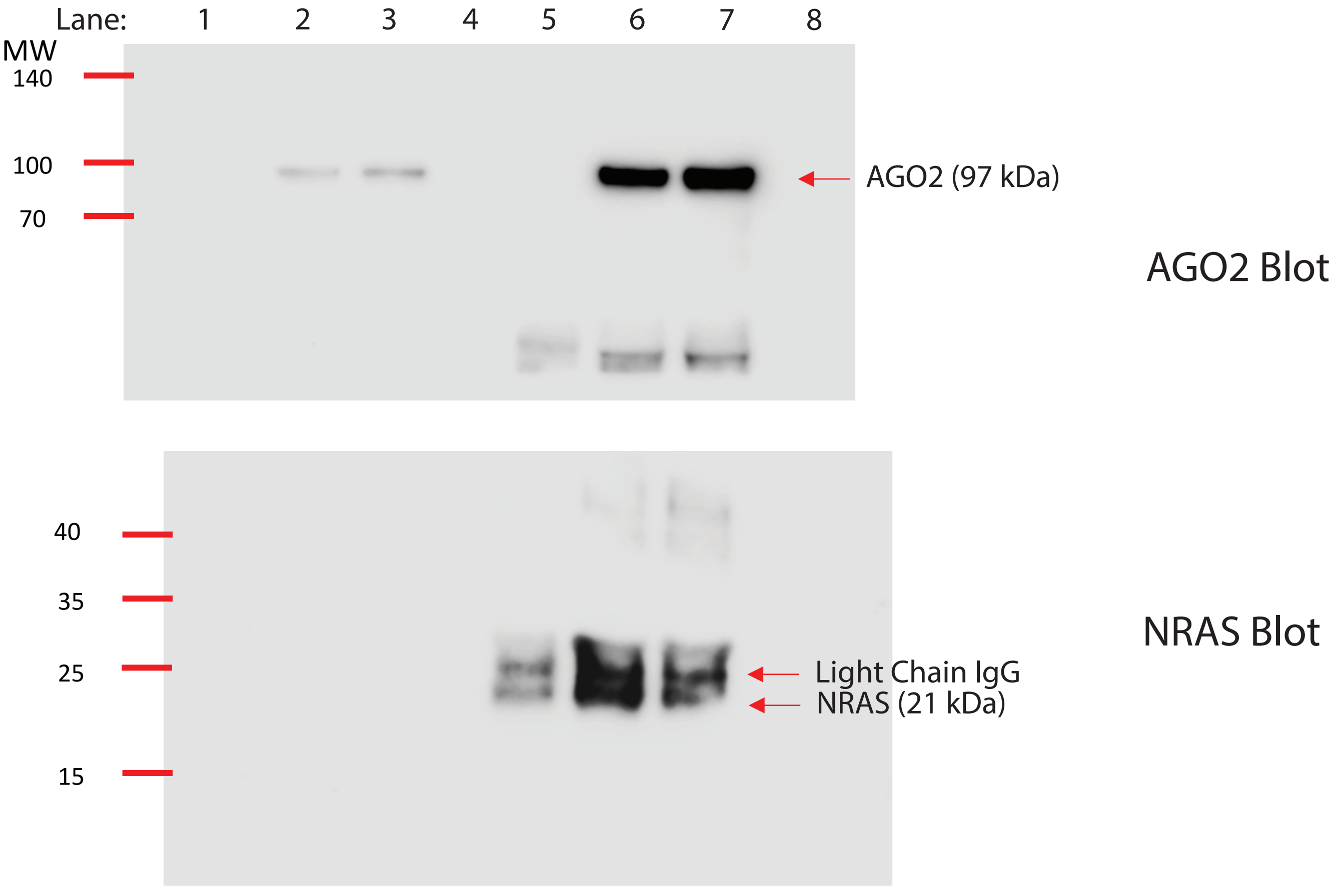

Lane Key:
1. Ladder
2. Serum Starve (5% Input Control)
3. Serum Starve + 5min EGF (5% Input Control)
4. Ladder
5. IgG Control
6. AGO2-IP (Serum Starve)
7. AGO2-IP (Serum Starve + 5min EGF)
8. Ladder

Figure R7 Raw western blot images from Figure S1. Uncropped raw western blot data at various exposures included in Supplemental Figure 1.

Raw Data From Supplemental Figure 1D: H1299 AGO2 Immunoprecipitation

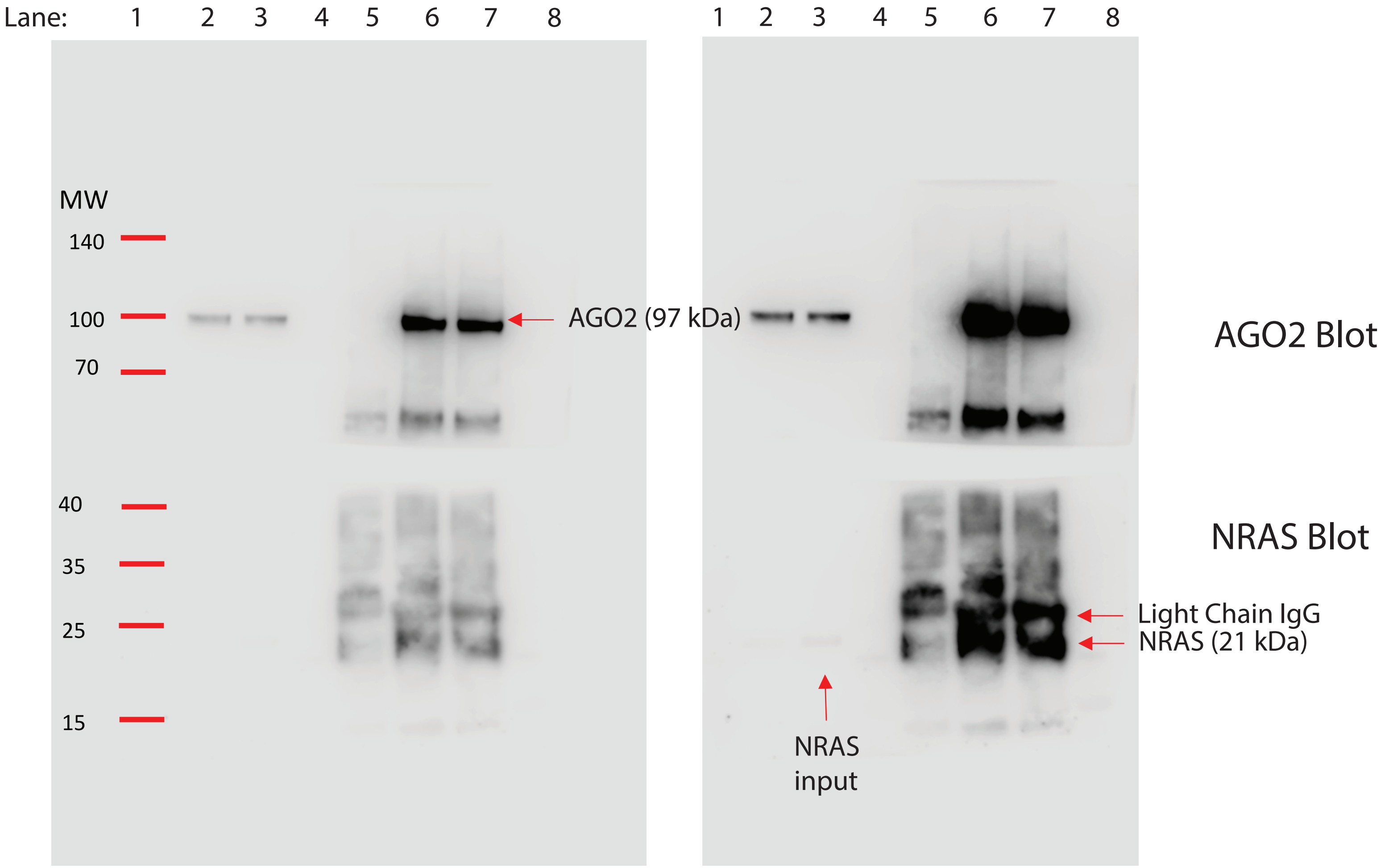

Lane Key:
1. Ladder
2. Serum Starve (5% Input Control)
3. Serum Starve + 5min EGF (5% Input Control)
4. Ladder
5. IgG Control
6. AGO2-IP (Serum Starve)
7. AGO2-IP (Serum Starve + 5min EGF)
8. Ladder

Figure R7 Raw western blot images from Figure S1. Uncropped raw western blot data at various exposures included in Supplemental Figure 1.
